# Unexpected XPS Binding Energy Observations Further Highlighted by DFT Calculations of Ruthenocene-Containing [Ir^III^(ppy)_2_(RCOCHCORc)] Complexes: Cytotoxicity and Crystal Structure of [Ir(ppy)_2_(FcCOCHCORc)]

**DOI:** 10.3390/molecules29225383

**Published:** 2024-11-15

**Authors:** Blenerhassitt E. Buitendach, Elizabeth Erasmus, Eleanor Fourie, Frederick P. Malan, Jeanet Conradie, J. W. (Hans) Niemantsverdriet, Jannie C. Swarts

**Affiliations:** 1 Department of Chemistry, University of the Free State, Bloemfontein 9300, South Africaerasmuse@ufs.ac.za (E.E.); fouriee@ufs.ac.za (E.F.); conradj@ufs.ac.za (J.C.); 2 Department of Chemistry, University of Pretoria, Pretoria 0002, South Africa; frikkie.malan@gmail.com; 3 Syngaschem, Valeriaanlaan 16, 5672 XD Nuenen, The Netherlands; jwn@syngaschem.com

**Keywords:** iridium, ferrocene, ruthenocene, β-diketonato complexes, X-ray photoelectron spectroscopy, DFT calculations, X-ray-induced decomposition, cytotoxic properties, antibacterial activity

## Abstract

The series of iridium(III) complexes, [Ir(ppy)_2_(RCOCHCOR′)], with R = CH_3_ and R′ = CH_3_ (**1**), Rc (**2**), and Fc (**3**), as well as R = Rc and R′ = Rc (**4**) or Fc (**5**), and R = R′ = Fc (**6**), ppy = 2-phenylpyridinyl, Fc = Fe^II^(η^5^–C_5_H_4_)(η^5^–C_5_H_5_), and Rc = Ru^II^(η^5^–C_5_H_4_)(η^5^–C_5_H_5_), has been investigated by single-crystal X-ray crystallography and X-ray photoelectron spectroscopy (XPS) supplemented by DFT calculations. Here, in the range of 3.74 ≤ Σχ_R_ ≤ 4.68, for Ir 4f, Ru 3d and 3p and N 1s orbitals, binding energies unexpectedly decreased with increasing Σχ_R_ (Σχ_R_ = the sum of Gordy group electronegativities of the R groups on β-diketonato ligands = a measure of electron density on atoms), while in Fe 2p orbitals, XPS binding energy, as expected, increased with increasing Σχ_R_. Which trend direction prevails is a function of main quantum level, n = 1, 2, 3…, sub-quantum level (s, p, d, and f), initial state energies, and final state relaxation energies, and it may differ from compound series to compound series. Relations between DFT-calculated orbital energies and Σχ_R_ followed opposite trend directions than binding energy/Σχ_R_ trends. X-ray-induced decomposition of compounds was observed. The results confirmed good communication between molecular fragments. Lower binding energies of both the Ir 4f_7/2_ and N 1s photoelectron lines are associated with shorter Ir-N bond lengths. Cytotoxic tests showed that **1** (IC_50_ = 25.1 μM) and **3** (IC_50_ = 37.8 μM) are less cytotoxic against HeLa cells than cisplatin (IC_50_ = 1.1 μM), but more cytotoxic than the free β-diketone FcCOCH_2_COCH_3_ (IC_50_ = 66.6 μM).

## 1. Introduction

**Figure 1:**
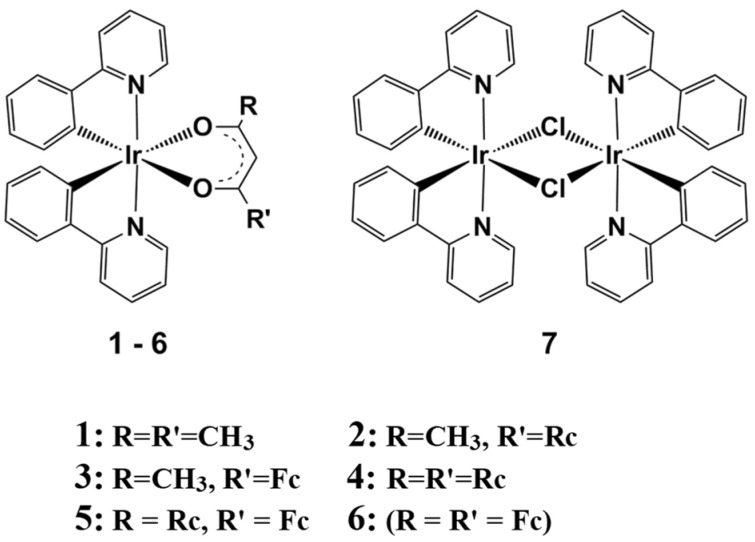
Structures of **1**–**6** as well as [Ir(ppy)_2_Cl]_2_, **7**. ppy = 2−phenylpyridinyl; Fc = Fe^II^(η^5^–C_5_H_4_)(η^5^–C_5_H_5_) = ferrocenyl; and Rc = Ru^II^(η^5^–C_5_H_4_)(η^5^–C_5_H_5_) = ruthenocenyl.

We recently described the synthesis of [Ir(ppy)_2_(H_3_CCOCHCOCH_3_)], **1**, as well as five new ferrocenyl- and ruthenocenyl-containing iridium(III) complexes [Ir(ppy)_2_(RCOCHCOR′)], **2**–**6**, with ppy = 2-phenylpyridinyl, ferrocenyl = Fc = Fe^II^(η^5^–C_5_H_4_)(η^5^–C_5_H_5_), and ruthenocenyl = Rc = Ru^II^(η^5^–C_5_H_4_)(η^5^–C_5_H_5_) by reacting appropriate metallocene-containing β-diketones with [Ir(ppy)_2_(μ-Cl)]_2_, **7** [1]. R and R′ are defined in Figure 1.

Only **1** is phosphorescent. Ferrocenyl and ruthenocenyl groups as well as oxidised Ir^IV^ centres quench all phosphorescence. The electrochemistry of these compounds was investigated [1], and it was found that the ferrocenyl group is oxidised at the lowest potentials, followed by the Ir^IV/III^ redox event and finally the ruthenocenyl Rc^+/0^ couple, all in an electrochemically reversible way. However, the highly reactive 17-electron ruthenocenium species, Rc^+^, interacted with the mother molecules as a whole and caused the Fc^+/0^ and Ir^IV/III^ couples to lose chemical reversibility during CV scans. The formal reduction potential of the Ir^IV/III^ couple, E°′(Ir^IV/III^), was linearly related [1] to the electron density of the β-diketonato ligand expressed as the sum of the Gordy group electronegativities [2] of the R and R′ β-diketonato groups (χ_R_ + χ_R′_ = Σχ_R_) in **2**–**6** with the equation E°′(Ir^IV/III^) = 0.2311 Σχ_R_—0.6554. We also reported the structure of **2** based on single-crystal X-ray diffraction [1].

The X-ray photoelectron spectroscopic (XPS) information of [Ir(ppy)_2_Cl]_2_, **7**, and [Ir(ppy)_2_(H_3_COCHCOCH_3_)], **1**, has been reported [3,4], but to our knowledge, no XPS study on any metallocene-containing [Ir(ppy)_2_(RCOCHCORʹ)] complex has hitherto been described. X-ray photoelectron spectroscopy identifies elements and their oxidation states in a sample and highlights the influence of the chemical environment on compound constituent elements. Group electronegativities also influence XPS-measured binding energies indicating that, in general, electron-withdrawing substituents increase the binding energy of atoms they are bound to [5]. Several XPS studies have been performed on non-iridium metallocene complexes such as [Mn(FcCOCHCOR)_3_] [5] and others [6,7,8]. One feature of ferrocene and its derivatives is that it has a propensity to become oxidised to ferricenium, Fc^+^, and thereafter to iron oxides under the influence of X-ray irradiation, especially in the presence of substrates bearing oxygen [9,10,11]. Reduction of the ferrocenyl iron under X-ray irradiation is not common. In contrast, only a few reports describing the XPS of the ruthenocenyl group in ruthenocene derivatives are available [12,13,14]. Most reports indicate that, as the ferrocenyl group, the ruthenocenyl species tends to oxidise to ruthenocenium upon irradiation during XPS experiments [12,13,14]. Ruthenocenyl XPS studies are complicated by a near-overlap of Ru 3d_5/2_ photoelectron lines with complex and adventitious carbon 1s photoelectron lines around 284.5 eV.

Iridium(III) complexes may have anticancer and antibacteriological applications. Towards this end, it was shown that [Ir(N,N′-dimethylurea)(cyclooctadiene)]Cl has antibacterial activity against Gram-positive as well as Gram-negative bacteria [15]. Other applications of iridium(III) complexes include biological imaging [16], use as electrochemical [17,18] or organic light-emitting devices [19,20], dye-sensitised solar cells [21,22], sensors [23,24], and as catalysts in, for example, the Cativa process in the generation of acetic acid from methanol [25,26,27]. It is also used as an electrocatalyst for oxygen evolution in water splitting [28,29,30]. Bimetallic oxides of ruthenium and iridium are also active in electrochemical oxygen generation from water [31,32,33].

The stability, reversible electrochemical behaviour, and ease of chemical synthesis of ferrocene [34,35,36], and to a lesser extent, of ruthenocene [37], allows them to be used as antineoplastic [38,39,40] and antimalarial materials [41], smoke suppressors [42,43], catalysts in chemical synthesis [44,45,46], and also high-burning-rate solid fuel catalysts [47].

In this study, we present the crystal structure of [(ppy)_2_Ir(FcCOCHCORc)], **5**, discuss the relationships between Ru, Ir, and N atom XPS binding energies (BEs) and electron density expressed as the sum of the Gordy group electronegativities of the R and R′ β-diketonato side groups, Σχ_R_, and compare these relationships with the Σχ_R_ vs. DFT-calculated orbital energy relationships of **1**–**6**. Relationships between previously determined redox potentials [1] (and also crystallographically determined bond lengths from known structures) and the present XPS results are also highlighted, and we report on the potential biomedical application of these complexes.

Normally. it is found that as the electron density on a specific atom is lowered (i.e., χ_R_ or Σχ_R_ is increased), that atom would exhibit larger XPS binding energies. Unique to our described results here is the result that, for Ir, Ru, and N atoms in our large molecules, as the χ_R_ or Σχ_R_ values are increased on these specific atoms, the XPS binding energies became smaller. This is basically unheard of in the literature, probably because practical research chemists do not know how to explain it and, therefore, may refrain from publishing it. In contrast to XPS binding energies, DFT-calculated orbital energies have negative signs and are therefore inversely related to XPS binding energies. Thus, although normally an increase in χ_R_ or Σχ_R_ should indicate a decrease in DFT-calculated orbital energies, here, the DFT-calculated results exhibit an unusual but internally consistent trend with XPS measurements, showing an increase in DFT-calculated energies of the appropriate Ru, Ir, and N orbitals with increasing χ_R_ or Σχ_R_.

## 2. Results and Discussion

### 2.1. Single-Crystal X-Ray Structure of ***5***

**Figure 2:**
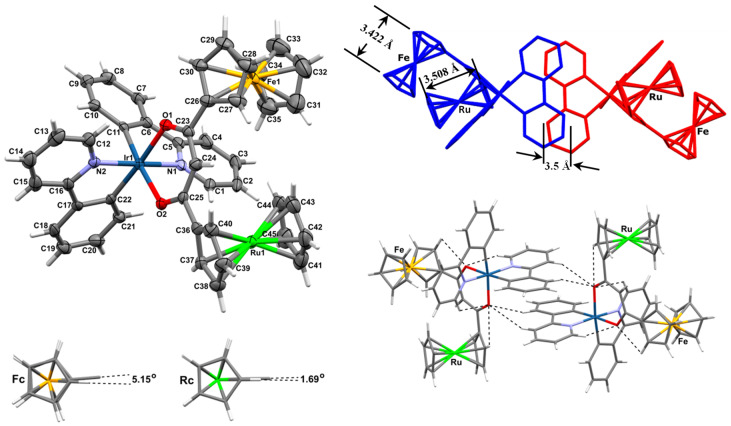
**Top left:** Molecular structure of [Ir(ppy)_2_(FcCOCHCORc)], **5**, showing atom labelling. H atoms coloured light grey are unlabelled. The thermal ellipsoids represent a 50% probability limit. **Bottom left:** The ferrocenyl cyclopentadienyl deviates further from an eclipsed conformation than the ruthenocenyl cyclopentadienyl rings. **Top right:** Crystal packing of neighbouring molecules of **5**, showing π-ring system overlap of ppy ligands. The π–π spacing between the phenylpyridinyl ligand planes of adjacent molecules is ca. 3.5 Å. **Bottom right:** Stabilising C–H···O interactions, indicated by black dashed lines, within **5**. Selected bond lengths (Å) relevant to the discussions below are the following: Ir(1)–N(1) 2.032(4), Ir(1)–N(2) 2.038(4), Ir(1)–C(11) 1.992(5), Ir(1)–C(22) 2.000(6), Ir(1)–O(1) 2.137(4), Ir(1)–O(2) 2.163(4), O(1)–C(23) 1.271(6), O(2)–C(25) 1.272(7), C(23)–C(24) 1.402(7), C(24)–C(25) 1.400(8), C(23)–C(26) 1.471(8), and C(25)–C(36) 1.481(7). Selected bond angles (degrees): N(1)–Ir(1)–C(11) 80.9(2), N(2)–Ir(1)–C(22) 80.6(2), O(1)–Ir(1)–O(2) 89.3(1), N(1)–Ir(1)–N(2) 174.1(2), O(1)–Ir(1)–C(22) 175.7(2), O(2)–Ir(1)–C(11) 173.8(2), Ir(1)–O(1)–C(23) 124.2(3), Ir(1)–O(2)–C(25) 123.6(3), O(1)–C(23)–C(24) 126.7(5), O(2)–C(25)–C(24) 127.0(5), C(23)–C(24)–C(25) 128.6(5), O(1)–C(23)–C(26) 115.0(4), and O(2)–C(25)–C(36) 115.3(4). Other bond lengths and angles are available in the Supplementary Materials. Symmetry transformations used to generate equivalent atoms: #1 −x, −y, −z.

The structure of [Ir(ppy)_2_(FcCOCHCORc)], **5**, was solved inter alia to relate bond lengths (as a measure of bond strength) with XPS binding energies. As **2** [1], complex **5** crystallised as orange blades from CH_2_Cl_2_/n-heptane in the monoclinic space group P 2_1_/n. Crystallographic data are given in Table 1 and a molecular plot of **5** is shown in Figure 2.

Complex **5** displayed a slightly distorted octahedral coordination geometry around the central Ir atom with angles N(1)–Ir(1)–N(2), O(1)–Ir(1)–C(22), and O(2)–Ir(1)–C(11) being 174.1(2), 175.7(2), and 173.8(2)°, respectively, rather than the expected 180.0°. Like **1** [48,49,50] and **2** [1], complex **5** exhibits the *cis*–C,C′ *trans*–N,N′ chelate disposition. Average Ir–N bonds (Ir–N_avg_ = 2.035 Å) are longer than average Ir–C bonds (Ir–C_avg_ = 1.996 Å) and correspond well with those of the precursor dimer [Ir(ppy)_2_(μ-Cl)]_2_ [51] and complexes **1** [48,49,50] and **2** [1].

The Ir(1)–O(1) and Ir(1)–O(2) bond lengths are 2.137(4) and 2.163(4) Å, respectively, with the slightly longer bond length on the ruthenocenyl side of the β-diketonato ligand. This is the result of the ruthenocenyl group being turned substantially out of the β-diketonato ligand plane defined by atoms O(1)–C(23)–C(24)–C(25)–O(2) by 28.17°. Utilising a 3σ(I) criterium, the Ir–O bond lengths are, however, the same, and so are the C(23)–C(24) (1.402(7) Å) and C(24)–C(25) (1.400(8) Å) bond lengths as well as bond lengths C(23)–O(1) (1.271(6) Å) and C(25)–O(2) (1.272(7) Å) (Figure 1), which implies that the β-diketonato ligand is symmetrically coordinated to the Ir(1) core and also that there is significant bond delocalisation in the β-diketonato molecular fragment coordinated to **5**.

Conjugated 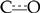 bond lengths in β-diketones have been reported to be about 1.27 Å, while conjugated 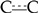 bond lengths are typically 1.37–1.42 Å [52]. Bond lengths and angles within the ppy ligand are typical for this ligand bound to Ir(III). The β-diketonato ligand bite angle O(1)–Ir(1)–O(2) for **5** was measured at 89.3(1)° compared to the 80.79° average of C(11 or 22)–Ir(1)–N(1 or 2) angles of the ppy ligands. This is the same pattern set by **1** [48,49,50] and **2** [1].

Regarding the Fc and Rc metallocenyl groups, positional disorder is displayed by the Fe and Ru atoms which occupy each other’s positions about 50% of the time. The average C–C bond distance within the ruthenocenyl group is 1.419 Å for the unsubstituted cyclopentadienyl (Cp) ring and 1.427 Å for the substituted Cp ring. The ferrocenyl group showed a slightly shorter average C–C bond distance at 1.413 Å for the unsubstituted Cp ring and 1.425 Å for the substituted Cp ring. The largest deviations from these averages are +0.022 Å for the C(31)–C(32) bond and −0.017 Å for the C(32)–C(33) bond. Bond angles in all four Cp rings averaged the ideal theoretical value for flat Cp rings, 108°. The largest deviation from the average values was for the C(31)–C(35)–C(34) angle (+1.95°) on the substituted Cp ring of the ferrocenyl group. The metallocenyl groups therefore exhibited normal delocalised bond lengths and angles.

The Cp rings of both of the metallocenyl groups were found to exist almost in the eclipsed conformation. Deviations from the eclipsed orientation were only 1.69° for the ruthenocenyl group (from the dihedral angles C(36)–(Cp-ring centroid)–(subst-Cp-ring centroid)–C(44)) but slightly more at 5.15° for the ferrocenyl group (C(26)–(Cp-ring centroid)–(subst-Cp-ring centroid)–C(35)), Figure 2. Cp–Cp-ring plane distances are 3.508 Å for the ruthenocenyl group and 3.422 Å for the ferrocenyl group.

Deviation of the Cp-ring planes from parallel to each other was measured at 0.77° for the ruthenocenyl group but was significantly larger for the ferrocenyl group at 3.03°. The ruhenocenyl-substituted Cp-ring plane deviated 28.17° from the β-diketonato chelate plane Ir(1)–O(1)–C(23)–C(24)–C(25)–O(2) but the equivalent ferrocenyl-substituted Cp-ring plane deviated only 11.63° from this β-diketone chelate plane. Both metallocenes project to the same side of the β-diketonato plane. These deviations and projection directions can clearly be seen in Figure 2, right, and are attributed to steric interference from the N1 ppy ligand and packing effects. In the absence of any steric interference or packing effects, these angles should be 0°.

The ppy-ligand positioning in the crystal packing of **5** is similar to that of **1**. A C2 rotation of neighbouring molecules positions a ppy ligand from one molecule nearly eclipsed relative to another ppy from an adjacent molecule; these ppy planes are ca. 3.5 Å apart as shown in Figure 2. Intermolecular π–π interactions between these ppy ligands are therefore possible with enhanced intermolecular communication as a result.

The crystals are stabilised by four intramolecular and two intermolecular C-H···O interactions (Figure 2). The four intramolecular C-H···O interactions include two ppy interactions between the atoms C(12)–H(12)···O(1) (H···O = 2.56 Å) and C(1)–H(1)···O(2) (H···O = 2.51 Å) as well as two metallocenyl C–H···O interactions between [C(30)–H(30)···O(1) (2.69 Å) and C(37)-H(37)···O(2) (2.79 Å)]. The two intermolecular C–H···O interactions arise as a three-centred bifurcated interaction of O2 with an adjacent ppy ligand with (C15–H15)_molecule A_···(O2)_molecule B_ (2.90 Å) and (C18–H18)_molecule A_···(O2)_molecule B_ (2.49 Å). These C–H···O interactions provide enhanced stabilisation of the overall structure.

### 2.2. X-Ray Photoelectron Spectroscopy

We previously [5] highlighted that the influence of the relative electron-withdrawing or electron-donating properties of R groups of free ferrocenyl-containing β-diketones FcCOCH_2_COR, R = CF_3_, C_6_F_5_, CH_3_, Ph = C_6_H_5_, and Fc as expressed by Gordy scale group electronegativities [2], χ_R_, regulates the position of the Fe 2p_3/2_ photoelectron line binding energy (BE) [5] in a way that may be quantified by the following linear equation:

BE (Fe 2p_3/2_) = 0.33χ_R_ + 707.41 (1)

The XPS of the β-diketone FcCOH_2_CORc (χ_Rc_ = 1.99 [53]) was, however, not included in that study. Equation (1) predicts that the XPS Fe 2p_3/2_ photoelectron line of FcCOH_2_CORc is located at BE(Fe 2p_3/2_) = 0.33(1.99) + 707.41 = 708.07 eV which corresponds well with the experimentally determined BE of 708.1 eV of this study. Supplementary Materials Figure S1 shows the Fe 2p XPS envelope of FcCOH_2_CORc. Although the errors on the binding energies were not determined, it is not considered to be more than 0.2 eV; data are provided to one decimal.

To investigate the structural and electronic consequences of the variation in the R groups within the β-diketonato ligands coordinated to Ir in [Ir(ppy)_2_(RCOCHCOR′)], XPS measurements were conducted on hydroxylated Si wafers spin-coated with **1**–**6**. Using a silicon wafer as a basis for studies has several advantages [54], not least because Si is a weak conductor and dissipates charge build-up of samples during XPS measurements well.

**Figure 3:**
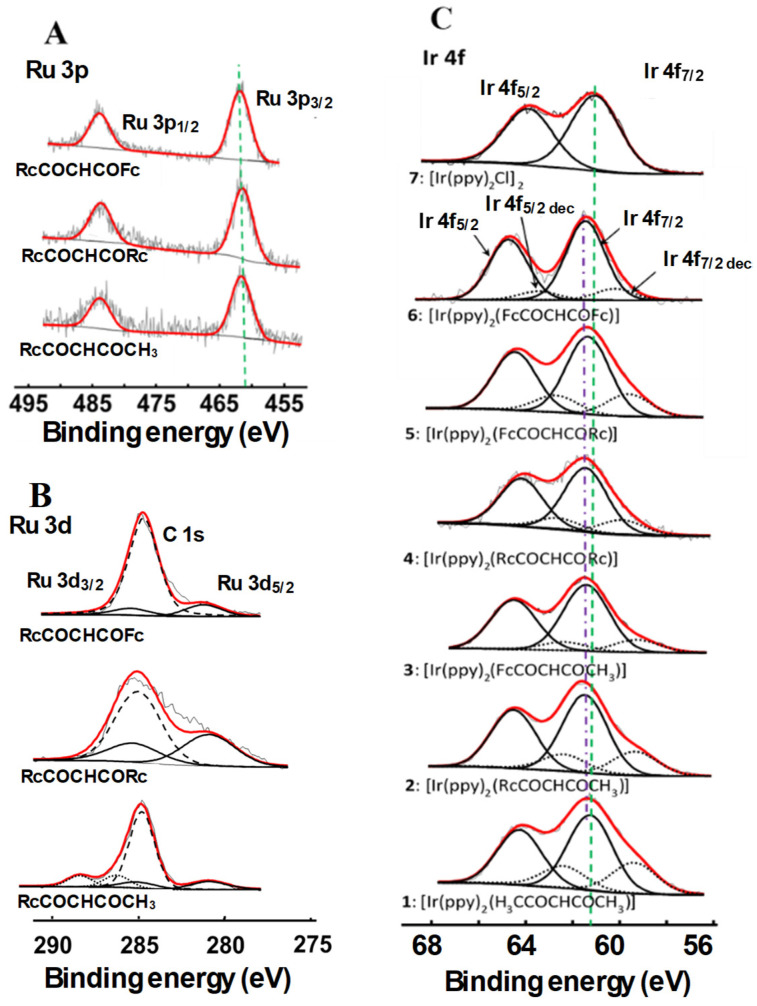
XPS spectra of (**A**) the Ru 3p and (**B**) Ru 3d region of free RcCOCH_2_COR β-diketones (R = CH_3_, Rc, and Fc), and (**C**) of **1**–**7** fitted with Gaussian-simulated Ir 4f_7/2_ and Ir 4f_5/2_ photoelectron lines as well as for the X-ray-induced decomposition products Ir 4f_7/2 dec_ and Ir 4f_5/2 dec_. The green vertical broken line (perpendicular to the X-axis) and purple broken line (following the Ir 4f_7/2_ photoelectron maximums) give an indication of binding energy shifts in moving from **1** to **6**. Notably, **7** showed no X-ray-induced decomposition.

**Figure 4:**
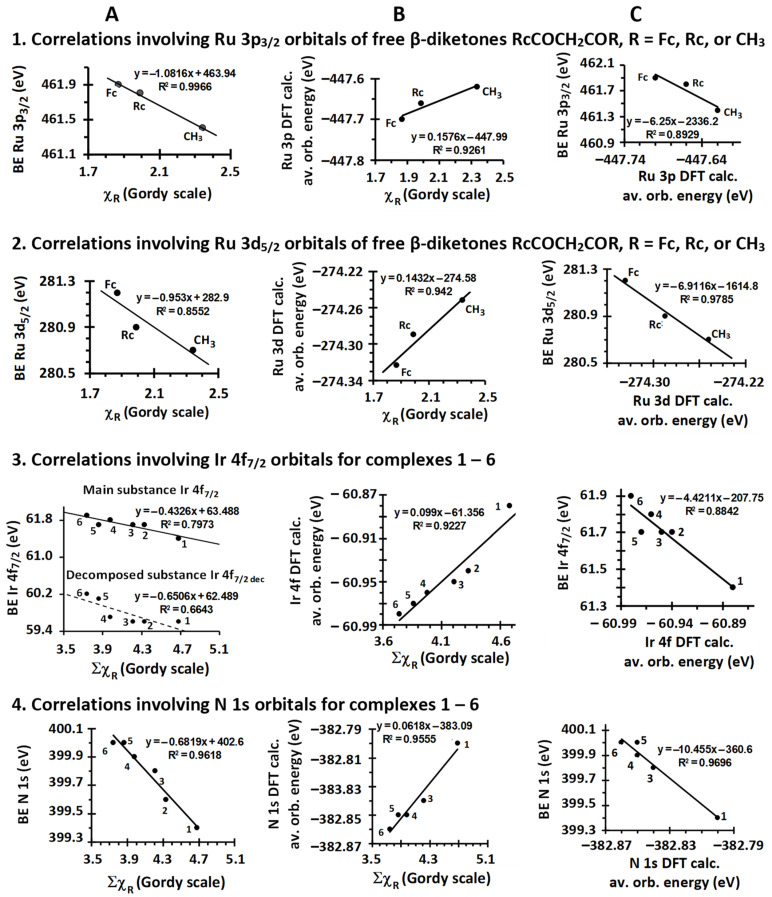
Column (**A**): The relationship between R group Gordy scale group electronegativities, χ_R_ (or the sum of β-diketonato R group electronegativities, Σχ_R_) and the binding energies (BEs) associated with the indicated photoelectron lines. Generally, measured binding energies are directly related to initial state properties such as group electronegativities of substituents on a compound if final state effects lead to roughly constant BE changes in a compound series, and one would expect the BE to increase when Σχ_R_ increases, but here, the trends are opposite. Deviations from this generalisation may be observed if the electronic structure in the initial configuration is influenced by the final state relaxation, leading to larger or smaller BE values, but the relationship is complex and, to our knowledge, not readily predictable. Column (**B**): The relationship between the indicated DFT-calculated orbital energies and χ_R_ (or Σχ_R_). By convention, orbital energies have opposite signs to XPS BEs, and hence, slopes opposite to those found in Column (**A**) are expected, but, because Column (**A**) relationships unexpectedly exhibited negative slopes, positive slopes are observed here. Generally, slopes in Column (**B**) would be expected to have a negative sign. Column (**C**): The expected inverse proportionality trend of plots of XPS BEs versus DFT-calculated orbital energies. In principle, a direct proportionality (i.e., positive slope) should never be obtained.

No Ru 3p_3/2_ photoelectron line BE—χ_R_ relationship has been investigated yet. In this study, three ruthenocenyl-containing β-diketones, RcCOCH_2_COR, with R = Fc, Rc, and CH_3_ became available. Ru 3p_3/2_ photoelectron lines of these β-diketones were therefore measured (Figure 3A) and a BE(Ru 3p_3/2_)—χ_R_ plot was constructed, Figure 4, Row 1, Column A); χ_R_ values are from Table 2. A least squares fit of data points gave Equation (2).

BE(Ru 3p_3/2_) = −1.08χ_R_ + 463.94; R^2^ = 0.997 (2)

In general, as the electron density on a specific atom is lowered (i.e., χ_R_ or Σχ_R_ is increased), that atom would cling to its core electrons strongly. In the absence of final state energy relaxation effects influencing BE measurements, this in almost all cases leads to larger XPS binding energies. Exceptions to this rule of thumb are known and notably include alkali metals such as potassium and caesium, where the XPS binding energies of these elements in a cationic form in chloride or fluoride matrixes are lower than in the metallic form. For these alkali metal cations, this is due to a dominant contribution of the Madelung potential as described by Siegbahn’s Charge Potential Model [55,56]. The Madelung potential refers to the electrostatic potential shift experienced by an atom in a crystal lattice due to the surrounding *charged* atoms in the unit cell. Complexes **1**–**6**, however, do not fall in this category as the remaining cationic component of **1**–**6**, after the release of a photoelectron during X-ray irradiation, is not embedded in a sea of anions. Rather, it is embedded in a sea of neutral atoms as the large overall structure of each complex is not charged, and this implies that Coulomb effects as applied to K^+^ embedded in a sea of Cl^−^, for example, does not play a role. The BE—Fe 2p_3/2_ relationship described in Equation (1) has, as expected, a positive slope with χ_R_ because the photoelectron BE increases as the electron density on an iron atom decreases under the influence of more electron-withdrawing ligands expressed as increasing ligand χ_R_ values. However, the Ru 3p_3/2_ relationship above has a *negative* slope (Equation (2)), indicating that BE *decreases* as the ligand χ_R_ increases.

This unexpected trend prompted us to also investigate the Ru 3d photoelectron lines even though they overlap with the carbon C 1s region, as shown in Figure 3B. By simulating the Ru 3d_3/2_ and Ru 3d_5/2_ photoelectron lines together with the C 1s photoelectron line and plotting the data in a BE(Ru 3d_5/2_)—χ_R_ relationship (Figure 4, Row 2, Column A), Equation (3) was determined.

BE(Ru 3d_5/2_) = −0.95χ_R_ + 282.00; R^2^ = 0.86 (3)

Again, an unexpected inverse trend as indicated by the negative slope was obtained. In contrast, the expected increase in BE(Ru 3d_5/2_) with increasing electron-withdrawing capability (i.e., χ_R_) of cyclooctadiene and pentamethylcyclooctadiene Ru^II^ ligands was described by Gassman and Winter [13]. However, these authors did not report on the relationship between BE(Ru 3p_3/2_) and electron-withdrawing properties of ligands associated with ruthenocenyl derivatives. These two result sets suggest that photoemissions from different elemental orbital levels (s, p, d, f) may exhibit unexpected trends and that first-, second-, and third-row metal complexes may behave differently than intuitively expected as described above.

In an attempt to verify the unexpected negative slope quantified by Equation (2) in the BE(Ru 3p_3/2_)—χ_R_ plot in Figure 4, Row 1, Column A, we also calculated the average 3p-π molecular orbital (MO) DFT energies of the Ru 3p orbitals of the free RcCOCH_2_COR β-diketones; they were found to be −447.62 (for R = CH_3_), −447.66 (R = Rc), and −447.70 eV (R = Fc). By convention, DFT-calculated orbital energies have negative signs and are inversely related to XPS BEs [57]. Thus, a plot of DFT-calculated Ru (3p-π)_ave_ orbital energies vs. χ_R_ should have a slope with an opposite sign to the slope of a BE(Ru 3p_3/2_)—χ_R_ plot, and a plot of experimental XPS BEs against DFT-calculated Ru (3p-π)_ave_ orbital energies should always have a negative slope. Upon plotting these two relationships, we obtained an unusual but expected (and thus internally consistent) positive slope for the plot of DFT-calculated energies of the Ru (3p-π)_ave_ orbitals vs. χ_R_ because it exhibited the opposite sign of the negative slope of the BE(Ru 3p_3/2_)—χ_R_ relationship; see Figure 4, Row 1, Columns B and A.

Furthermore, the slopes of the diagrams in columns A and B would be expected to be about the same numerically but with opposite signs if DFT theory and the basis sets and functionals we used compensated in the same way as XPS BE calculations for different energy relaxation effects and resonance contributions. The observed slopes in Figure 4, Row 1, Columns B and A, were, however, +0.1576 and −1.0816, respectively, which are mutually consistent in opposite-sign slope trend predictions but not in accurately calculated numbers. 

Measured binding energies are directly related to initial state properties such as group electronegativities of substituents on a compound if final state effects lead to roughly constant BE changes. This is consistent with the positive slope of Equation (1) but not the negative slopes in Equations (2) and (3). Deviations from this generalisation may be observed if the electronic structure in the initial configuration is influenced by the final state relaxation in a non-constant way, leading to larger or smaller BE values, but the relationship is complex and, to our knowledge, not readily predictable. A good guide on relaxation effects is available elsewhere [58]. We recently reported another example of such a deviation with gold complexes [59]. It is concluded that different final state energy relaxation effects are the cause of the unexpected negative slopes of Equations (2) and (3) and the large numerical differences in DFT-calculated orbital energies and XPS-observed experimental binding energies.

The expected negative slope for the plot of the experimental XPS BE vs. the DFT-calculated Ru 3p orbital energy is shown in Figure 4, Row 1, Column C, but the numerical slope of −6.25 is much larger than the expected slope of −1, probably for the same final state relaxation energy reasons described above.

The same trends were also observed for the Ru 3d_5/2_ orbitals. DFT-calculated energies also increased with χ_R_ (see the DFT-calculated Ru (3d-t_2g_)_ave_ orbital energies vs. the χ_R_ plot in Figure 4, Row 2, Column B) and are, as expected, opposite in trend direction to that of the BE(Ru 3d_5/2_)—χ_R_ plot (Figure 4, Row 2, Column A). The numerical slope values of these two plots, −0.953 and +0.1432, respectively, also differed greatly from each other, and the negative slope of −6.9116 for the BE(Ru 3p_3/2_)—(DFT-calculated Ru (3p-π)_ave_ orbital energies) plot (Column **C**, Row 2, Figure 4) deviated substantially from −1, probably for the same energy relaxation energy reasons proposed above in the Ru (3p-π)_ave_ discussion.

To further investigate these unexpected trends, the third-row transition metal Ir, second-row transition metal Ru, and first-row transition metal Fe as well as the main group N atoms of the neutral coordination complexes **1**–**6** were also subjected to an XPS and DFT investigation utilising s, p, d, and f orbitals. The results are described below.

The XPS of the Ir 4f_7/2_ photoelectron line of compound **7**, [(ppy)_2_IrCl]_2_ (binding energy = 61.4 eV), was found to be at the same binding energy position as reported by Polosan et al. [3] but at a lower binding energy position than the new β-diketonato complexes **1**–**6**. Additionally, **7** shows its nitrogen N 1s photoelectron line at 399.6 eV, within the same binding energy range as that observed for **1**–**6** (see below). The chlorine Cl 2p photoelectron lines are at 198.2 and 199.9 eV (for Cl 2p_3/2_ and Cl 2p_1/2_, respectively). Complexes **1**–**6** do not possess Cl atoms.

The XPS spectra of **1**–**7** (Figure 3C) show the binding energy associated with the Ir 4f photoelectron lines; data from these spectra are presented in Table 2. Two distinct sets of peaks were simulated for the Ir 4f photoelectron lines. The main set of peaks is associated with complexes **1**–**6** while the second extra photoelectron line, set at lower energies, is associated with X-ray-induced decomposition products of **1**–**6**. The range in which the compound Ir 4f_7/2_ photoelectron lines is found, 61.4 ≤ BE(Ir 4f_7/2_) ≤ 61.9 eV, spans 0.5 eV. The smaller decomposition product photoelectron lines are found at the lower energy side between 59.2 and 60.2 eV, as shown in Table 2.

**Table 2:** The sum of R group Gordy scale group electronegativities, Σχ_R_, electrochemically measured reduction potentials of the Ir^III/IV^ redox wave, and Ir 4f_7/2_ compound and decomposition product Ir 4f_7/2_ as well as N 1s binding energies, available crystallographically determined average Ir-N bond lengths, and the indicated element DFT-calculated orbital energies of **1**–**6**.

	R(χ_R_)	R′(χ_R′_)	Σχ_R_ [1] ^a^	Ir^III/IV^ E°′ (V)	BE Ir (eV)			I_ratio_ ^b^	(Ir−N)_av_	DFT Orb. En. (eV)	
					Ir 4f_7/2_	Ir 4f_7/2,dec_	N 1s		(Å)	Ir 4f (t_2u_ + a_2u_)_ave_	N 1s
**1**	CH_3_(2.34)	CH_3_	4.68	0.319	61.4	59.2	399.4		2.010 [48,49,50]	−60.88	−382.80
**2**	Rc(1.99)	CH_3_	4.33	0.283	61.7	59.2	399.6	0.28	2.047 [1]	−60.94	
**3**	Fc(1.87)	CH_3_	4.21	0.497	61.7	59.2	399.8	0.32		−60.95	−382.84
**4**	Rc(1.99)	Rc	3.98	0.252	61.8	59.3	399.9	0.19	2.033 [60]	−60.96	−382.85
**5**	Fc(1.87)	Rc	3.86	0.445	61.7	60.1	400.0	0.37	2.035 ^c^	−60.97	−382.85
**6**	Fc(1.87)	Fc	3.74	0.681	61.9	60.2	400.0	0.22		−60.98	−382.86
**7**	[(ppy)_2_IrCl]_2_				61.3						

^a^ By way of example, for **2**, Σχ_R_ is calculated as follows: Σχ_R_ = χ_Rc_ + χ_CH3_ = 1.99 + 2.34 = 4.33. ^b^ I_ratio_ = ratio between the intensities of the decomposition and main product Ir 4f_7/2_ photoelectron lines [=(I_Ir 4f7/2 dec_)/(I_Ir 4f7/2_)]. ^c^ This work.

To understand the origin of the decomposition peaks, in a separate experiment, complex **6** was irradiated for more than 48 h and the XPS spectra were recorded at different times. After 5 min. of irradiation time, the decomposed product photoelectron lines were not yet detectable. However, after ca. 48 h, the original Ir 4f photoelectron peaks were barely detectable anymore and were replaced by those of the decomposition product. The Fe 2p and Ru 3p electron lines also shifted by more than 1.3 eV. It is well known that exposure to X-rays during XPS analysis may cause radiation-induced damage [61,62,63,64] that may include chemical or structural changes, compound decomposition, or even reduction of the metals [64]. As the Ir, Fe, and Ru decomposition peaks are separated from the main peak by more than 1.3 V (Table 2 and Table 3; in some Ru cases, it is even 4.1 eV), because the instrumental accuracy on measured binding energies is better than 0.2 eV and can resolve peaks with suitable software (we use Multipak version 8.2c computer software) within 0.5 eV, and because the percentage of decomposition never exceeded 37% after 17 min of exposure time (I_ratio_ values in Table 2), compound decomposition is not considered to influence the main peak BEs reported in this study.

**Figure 5:**
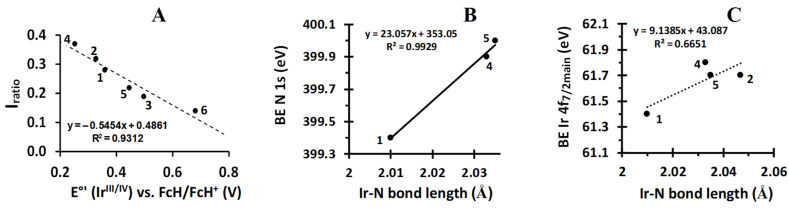
(**A**): Relationship between the electrochemically determined reduction potential of the Ir^III^/Ir^IV^ redox couple (E°′) and the I_ratio_ of Ir 4f_7/2_ photoelectron lines of **1**–**6** (I_ratio_ is the ratio between decomposition product and main product Ir 4f_7/2_ photoelectron line intensities). (**B**,**C**): Correlation of available Ir-N bond distances of **1**, **2**, **4**, and **5** with the N 1s binding energy (**B**) (**2** is omitted in graph (**B**) because it does not fit the trend line at all) and Ir 4f_7/2_ main photoelectron line (**C**).

The I_ratio_ entity (Table 2) is the ratio between the intensities of the decomposed and main product Ir 4f_7/2_ photoelectron lines (=(I_Ir 4f7/2 dec_)/(I_Ir 4f7/2_)) or otherwise stated, the fraction of X-ray-induced decomposition (radiation-induced damage) present in a sample, here, after 17 min of irradiation. The higher the value of I_ratio_, the more decomposition occurred. Inspection of Table 2 and Figure 5A shows that as the iridium formal reduction potential, E°′(Ir^III/IV^), increased, the quantity of I_ratio_ decreased. This indicates that the more difficult it is to oxidise the iridium within a sample, the less radiation-induced damage due to X-ray exposure can be observed. No relationship exists between Ir 4f_7/2_ binding energies and E°′(Ir^III/IV^).

Figure 4, Row 3, Column A, again, unexpectedly shows that the binding energy of the Ir 4f_7/2_ photoelectron lines for compounds **1**–**6** (as well as for the decomposition products of these complexes) decreases with increasing Σχ_R_ of the R groups on the β-diketonato ligand. This contrasts the expected, opposite trends observed numerous times before, for example, for the first-row metals Mn [65], Cu [66], Co [67], Cr [68], and Fe [65]. As with the free ligand Ru plots above, the plot of DFT-calculated Ir 4f (t_2u_ + a_2u_)_ave_ orbital energies versus Σχ_R_ highlighted the increase in orbital energies with increasing Σχ_R_ (Figure 4, Row 3, Column B), but the numerical absolute values of these slopes differed once again greatly (slopes are −0.4326 in Column A and 0.099 in B, respectively), and, although the XPS BE(Ir 4f_7/2_)—(DFT-calculated 4f (t_2u_ + a_2u_)_ave_ orbital energies) plot exhibited the expected negative slope, −4.4211, it deviated again substantially from negative unity, as seen in Figure 4, Row 3, Column C.

The N 1s photoelectron line of **1**–**6** was located at an average binding energy of ca. 399.8 eV in the range 399.4 ≤ BE(N 1s) ≤ 400.0 eV (Table 2); see Supplementary Materials Figure S2 for the spectra. No X-ray damage was apparent in the N 1s photoelectron lines. The measured binding energies are much larger than the 396.6 eV reported for the N of pyrrole-functionalised Fischer carbenes [69] but comparable with the average ca. 399.8 eV of the inner nitrogen (-C-N=C-) of the pyrrole moieties within porphyrin derivatives [70]. It appears that N binding energies differ greatly depending on the type of molecular structure they are part of. To explain these large differences, it has been reported that the binding energy of the N 1s photoelectron line decreases as the formal M-N bond order increases (M = metal) [71] and, by implication, the bond strength. There is also an interdependence between the contraction of the M-N bond and the shift in binding energies to smaller electron volts (eV). We observed a slightly larger than usual N 1s FWHM for our compounds (ca. 2.1 eV versus 1.8 eV for BN) [72]. Although the two N atoms in our molecules may contribute slightly to the observed increase in FWHM, the increase is more probably due to the consequence of utilising high-pass energy to achieve distinct peaks (consistently set at 93.90 eV for all elements) to compensate for the low X-ray power (25 W) to minimise compound damage.

In the case of **1**–**6**, the Ir-N bond order is always one; thus, an increase in the binding energy of the N 1s photoelectron line is due to the decrease in the bond strength (represented by the increase in bond length), as shown in Figure 5B. Our results show this concept can also be applied to the Ir 4f_7/2_ main photoelectron line. As the binding energy of the Ir 4f_7/2_ main photoelectron line decreases, the average Ir-N bond length also decreases; see Figure 5C. Since bond length is inversely proportional to the bond strength, it can be concluded that as the binding energy of the Ir 4f_7/2_ main photoelectron line decreases, the bond strength increases. This crystallographically based result for N and Ir is mutually consistent with the negative slope of the relationship between BE(Ir 4f_7/2_) and Σχ_R_ and also between BE(N 1s) and Σχ_R_ as shown in Figure 4, Row 3, Column A, and Row 4, Column A, respectively, despite the fact that N is a main group non-metallic element and Ir is a third-row transition metal. A positive slope of 0.0618 for the plot of DFT-calculated N 1s orbital energies vs. Σχ_R_, Figure 4, Row 4, Column B, was found, but this slope is more than 10 times smaller than the absolute value of 0.6829 for the BE(N 1s)—Σχ_R_ slope. The expected negative slope for the relationship between BE(N 1s) and DFT-calculated N 1s orbital energies is demonstrated in the Figure 4, Row 4, Column C, plot. Once again, the slope of −10.455 deviated greatly from negative unity. The described slope deviations from the expected trends of these three diagrams are again attributed to different relaxation energy effects as described above for Ru and Ir in large molecules such as **1**–**6**.

**Figure 6:**
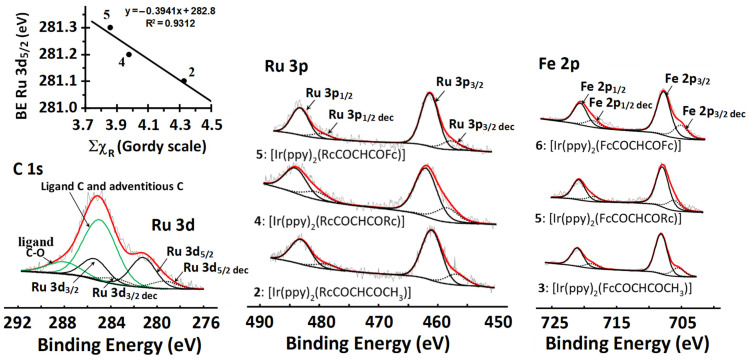
**Left top**: The Ru 3d_5/2_—Σχ_R_ relationship. **Left bottom**: The XPS spectrum for **5** showing the overlapping regions of the C 1s and Ru 3d photoelectron lines. **Middle**: Comparative XPS spectra of the Ru 3p area fitted with Gaussian-simulated peaks for **2**, **4**, and **5** and, to lower energy values, the decomposition-simulated Ru 3p_3/2_ and Ru 3p_1/2_ photoelectron lines. **Right**: Comparative XPS spectra of the Fe 2p area fitted with Gaussian-simulated peaks for **3**, **5**, and **6** as well as decomposition product-simulated Fe 2p_3/2_ and Fe 2p_1/2_ photoelectron lines. Remark: These Fe decomposition peaks are at such unusually low BEs that we suggest that they may be attributed to nonconductive decomposition products that cause charge build-up. This may result in loose material not in good electrical contact with the rest of the sample, implying that all the observed decomposition peaks may have artificial peak positions with undetermined charged shifts.

The XPS data of **1**–**6** with respect to the Ru 3p, Ru 3d/C 1s, and Fe 2p photoelectron line areas are summarised in Table 3, and the XPS spectra are shown in Figure 6. The Ru 3p_3/2_ photoelectron peaks of complexes **1**–**6** are in the range of 461.3 ≤ BE(Ru 3p_3/2_) ≤ 462.1 eV. These binding energies are slightly lower than the reported data for ruthenocenyl groups (462.4 eV) covalently anchored via silane linkers onto a Si wafer [12]. The Ru 3d_5/2_ photoelectron lines are situated between 281.1 and 281.3 eV, as shown in Table 3. Just as with Ir 4f, the XPS spectra of Fe 2p, Ru 3p, and Ru 3d of **1**–**6** all showed a decomposition peak at lower binding energies than that of the original compound, indicating that X-ray damage also occurred for Fe and Ru (Figure 6 and Supplementary Materials Figure S3).

No meaningful relationships could be established between the binding energies of the Ru 3p_3/2_ XPS peaks and Σχ_R_ or E_pa,Rc_, nor any of the relationships involving Ru 3p or 3d DFT-calculated orbital energies (Supplementary Materials Figure S4). Only the BE(Ru 3d_5/2_)—Σχ_R_ inverse proportionality could be estimated; see Figure 6, top left.

The Fe 2p_3/2_ binding energy of the ferrocenyl moiety was located in the range of 707.2 to 708.0 eV. The iron in a ferrocenyl group is a first-row transition metal Fe^II^ centre and the Fe 2p_3/2_ binding energy is much lower than the equivalent BE for Fe^II^ in hexacyanoferrates (708.5 eV) [73]. However, this relatively “low” binding energy value for Fe 2p_3/2_ in ferrocene is normal and correlates very well with other reported binding energy positions of the ferrocenyl group within other compounds at ca. 707.8 eV [5,6]. 

**Figure 7:**
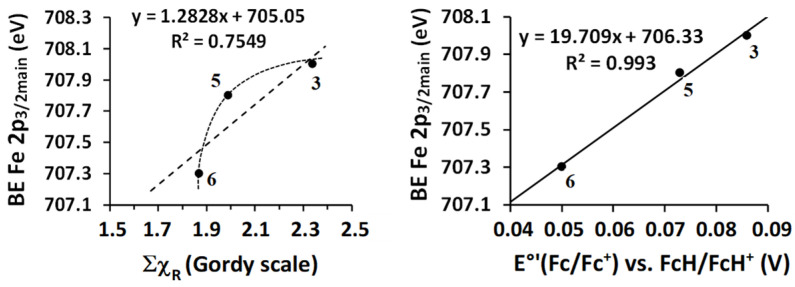
The BE of Fe 2p_3/2_ photoelectrons increased, as expected, with increasing E°′ linearly. The BE Fe 2p_3/2_—χ_R_ relationship also increases in the expected way, although the relationship is not linear. This trend direction is opposite to what was found for Ir, Ru, and N.

The relationships between BE(Fe 2p_3/2_) and χ_R_ as well as E°′(Fc/Fc^+^) of **3**, **5**, and **6** are shown in Figure 7. The trend of the non-linear BE(Fe 2p_3/2_)—χ_R_ plot indicates that the results are in accordance with general expectations, but opposite to what was found for Ru, Ir, and N, where the BE increases with increasing χ_R_ in the 1.88 ≤ χ_R_ ≤ 2.34 region. The linear relationship between BE(Fe 2p_3/2_) and formal reduction potentials of the Fc/Fc^+^ redox couple of **3**, **5**, and **6** (Figure 7, right) indicate a direct proportionality between Fe 2p_3/2_ photoelectron binding energy and the energy required for the electrochemical removal of an outer electron from the HOMO (highest occupied molecular orbital) of the ferrocenyl group. Both χ_R_ and E°′ (Fe^II/III^) are associated with initial state energy levels, and the observed plots in Figure 7 are consistent with final state energy relaxation having no or very little influence on the Fe 2p_3/2_ binding energies.

No meaningful relationship between BE(Fe 2p_3/2_) and DFT-calculated orbital energy for Fe 2p photoelectrons could be found (Supplementary Materials Figure S5).

Having characterised complexes **1**–**6** crystallographically here and elsewhere [1,61] electrochemically and spectroelectrochemically [1], spectroscopically [1] by ^1^H NMR, UV/vis, phosphorescence, and also with XPS and DFT (this work), applications of these new complexes were considered. Two applications are important from our point of view. Here, we report on biomedical applications of **1**–**6**. Of particular interest is to establish if there is a synergistic effect between the Ir core and the ferrocenyl centre in its antineoplastic activity. Such a synergistic effect was observed between a Rh^I^ centre and the ferrocenyl group. In a follow-up paper, we shall report on these complexes as electrocatalysts in the electrochemical splitting of water to generate oxygen gas.

### 2.3. Biomedical Properties

Complexes **1**–**6** were tested for antibacterial properties by performing a zone inhibition experiment utilising the plate diffusion technique [15] and a panel of antimicrobial-susceptible and -resistant Gram-positive *Staphylococcus aureus* and Gram-negative *Escherichia coli* and *Pseudomonas aeruginosa* bacteria. No zones of inhibition were observed. As we did not see any evidence of antibacterial activity, we have not conducted an MIC test. This result contrasts, for example, the activity of [Ir(N,N′-dimethylurea)(cyclooctadiene)]Cl which showed good antibacterial activity against both Gram-positive and Gram-negative bacteria [15].

**Figure 8:**
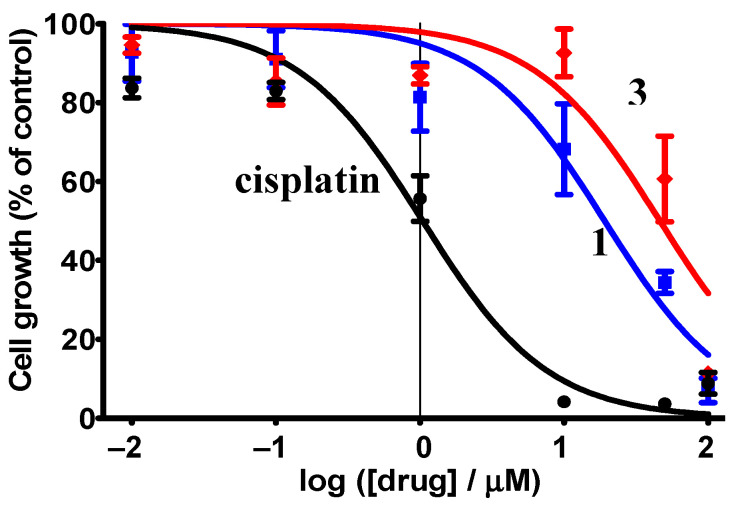
Effect of [Ir(ppy)_2_(FcCOCHCOCH_3_)] (**3**, red), [Ir(ppy)_2_(CH_3_COCHCOCH_3_)] (**1**, blue), and cisplatin concentration on the cell growth inhibition of HeLa cells incubated for 3 days from triplicate experiments.

Cytotoxic tests of [Ir^III^(ppy)_2_(CH_3_COCHCOCH_3_)], **1**, and [Ir^III^(ppy)_2_(FcCOCHCOCH_3_)], **3**, performed on the Cellonex cervical cancer HeLa cell line showed that these complexes exhibit antineoplastic activity. Cell survival curves (Figure 8) show cell growth expressed as a percentage of the control’s growth (in essence, this is cell growth inhibition) as a function of log(drug concentration in μM).

IC_50_ values, which are the mean concentration needed for 50% inhibition of cell growth, were obtained from three triplicated experiments and estimated by extrapolation; they are summarised in Table 4. The cytotoxicity of cisplatin was also determined for comparative purposes.

The obtained IC_50_ values illustrate the potential of these Ir(III) complexes as anticancer drugs and contrast the traditional view that iridium(III) is not cytotoxic due to a lack of reactivity [74]. Complexes **1** (IC_50_ = 25.1 μM) and **3** (IC_50_ = 37.8 μM) are less cytotoxic than cisplatin (IC_50_ = 1.1 μM under our conditions). [Ir^III^(ppy)_2_(R-Im-CH_2_Im-R)] complexes (Im = imidazole and R = methyl, ethyl, or butyl) have been found to be viable for photodynamic therapy (PDT) due to the photoluminescent properties they possess [75]. Since **3** does not possess photoluminescence due to the presence of the ferrocenyl group [1], it cannot be considered for PDT. However, complex **1** is photoluminescent [1] and is therefore also a potential candidate for PDT. Regarding [Ir^III^(ppy)_2_(FcCOCHCOCH_3_)], **3**, the IC_50_ value of 37.8 μM is significantly better than the IC_50_ value of 66.6 μM reported for the free β-diketone, FcCOCH_2_COCH_3_ [76].

The cytotoxic properties of the ferrocenyl core result, firstly, from its capability to reduce the tyrosyl radical of the R2 subunit of the enzyme ribonucleotide reductase (RNR). RNR catalyses the formation of 2′-deoxyribonucleotides from the four different ribonucleoside diphosphates, a reaction essential to DNA synthesis [77]. Reduction of the tyrosyl radical inactivates this enzyme and disrupts DNA synthesis in cancer cells. Mechanistically, therefore, the ferrocenyl mode of action involves electron transfer processes.

Secondly, ferrocenyl-containing drugs were also found to be cytotoxic if their redox potentials were low enough that they could be oxidised by redox-active body enzymes to ferrocenium fragments [78]. This Fe^III^ species then interacts with water and oxygen to generate a hydroxyl radical which cleaves DNA strands, ultimately leading to cell death [78]. The redox potential of the ferrocenyl group of [Ir(ppy)_2_(FcCOCHCOCH_3_)], **3** (E^o^ʹ = 0.086 V vs. FcH/FcH^+^), is smaller than that of the free β-diketone, FcCOCH_2_COCH_3_ (E^o^ʹ = 0.234 V vs. FcH/FcH^+^), which explains at least in part why **3** is more cytotoxic than free FcCOCH_2_COCH_3_. However, the iridium core must also play a cytotoxic role as H_3_CCOCH_2_COCH_3_ is not cytotoxic, but [Ir(ppy)_2_(H_3_CCOCHCOCH_3_)], **1**, is (Table 4). At this stage, it is not clear whether the cytotoxicity from the Ir^III^ core results from electron transfer processes involving Ir^III^ or, for example, intercalation into DNA strands. Further research is required to address this question.

## 3. Experimental Section

### 3.1. Compounds

Complexes **1**–**6** were prepared as described before by reacting [1] **7** with an appropriate β-diketone after the latter was separated from the aldol self-condensation product of acetyl ferrocene, FcCOCH=C(CH_3_)Fc [79].

### 3.2. Crystal Structure Determination of [Ir(ppy)_2_(FcCOCHCORc)], ***5***

Single-crystal diffraction studies on complex **5** were carried out using Quazar multi-layer optics monochromated Mo Kα radiation (k = 0.71073 Å) on a Bruker D8 Venture kappa geometry diffractometer (Bruker, Johannesburg, South Africa) with duo Iμs sources, a Photon 100 CMOS detector and APEX II control software (v2015.5-2) [80]. Measurements of X-ray diffractions were made at 150.0(2) K. SAINT+ (v8.34A) [80] was used for the data reduction while SADABS (v2014/2) [80] corrected the intensities for absorption. Direct methods were used to solve the structures by SHELXT (v2013/4) [81], using the SHELXL-2014/7 (v2014/7) [82] programme. The atoms other than hydrogen were refined anisotropically. Geometrically idealised positions were used for all H atoms which were constrained to ride on their parent atoms. Tables S1–S4 in the Supplementary Materials contains the data collection and refinement parameters as well as bond lengths and bond angles of the structure.

### 3.3. X-Ray Photoelectron Spectroscopy

A PHI 5000 Versaprobe spectrometer (Ulvac-Phi, Chigasaki, Japan) was used to record the X-ray photoelectron spectroscopy (XPS) data. A 50 µm diameter monochromatic Al Kα X-ray source (1486.6 eV) was generated by exposing an aluminium anode to a 25 W, 15 kV electron beam. The survey scans were recorded at a constant pass energy of 187.85 eV, and the detailed region scans were recorded at a constant pass energy of 93.90 eV, with the energy per step being 0.1 eV. The background pressure was 2 × 10^−7^ Pa. Other experimental details used during the experiments were the same as reported previously [5,65]. Although the errors on the binding energies were not determined, it is not considered to be more than 0.2 eV; data are provided to 1 decimal.

### 3.4. DFT Calculations

All complexes were optimised in the gas phase with scalar relativistic DFT calculations using the ADF [83] 2019 programme with all-electron ZORA-STO-TZ2P basis sets and the OLYP functional [84,85] including Grimme’s D3 dispersion correction [86]. From the output files of the DFT calculations, the MO energies of the N 1s, Fe 2p, Ru 3p, Ru 3d, and Ir 4f orbitals were obtained. For the p MOs, the two lowest energy MOs (of the three p MOs) were associated with the p-π orbitals and related to the p_3/2_ XPS binding energies. For the d MOs, the three lowest energy MOs (of the five d MOs) were associated with the t_2g_ orbitals of a molecule in an octahedral ligand field [87]. The average energy of the three t_2g_ orbitals, (3d-t_2g_)_ave_, was related to the Ru 3d_5/2_ XPS binding energies. For the f MOs, the four lowest energy MOs (of the seven f MOs) were associated with the triply degenerate t_2u_ and the a_2u_ orbitals of a molecule in an octahedral ligand field [87]. The average energy of the four orbitals, (t_2u_ + a_2u_)_ave_, was related to the Ir 4f_7/2_ XPS binding energies.

### 3.5. Cytotoxicity Measurements

Cell culture: The Cellonex human cervical cancer cell line (Hela cells) (Separations, South Africa) was grown in DMEM (Dulbecco’s modified essential medium), supplemented with 10% foetal calf serum and 1% penicillin/streptomycin. The cells were incubated in a humidified atmosphere with 5% carbon dioxide present at 37 °C. All serums, growth media, supplements, and other chemicals used in the cell culture work were purchased as sterile from Thermo Fisher Scientific, Pretoria, South Africa.

Cytotoxicity assay: Tests were performed according to the SRB (SRB = Sulforhodamine B) assay [88]. A monolayer of cells was trypsinised and suspended in a 1 mL growth medium. The cell count was adjusted to 0.5 × 10^5^ cells/mL. The diluted cell suspension (0.1 mL) was added to each well of a 96-well microplate. After adherence of the cells to the plate, 0.1 mL of different concentrations (Figure 8) of the test compounds was added to each well. The plates were incubated for 3 days at 37 °C with 5% CO_2_. A total of 0.05 mL of 50% trichloroacetic acid was added to each well, and the plates were incubated overnight at 4 °C. The plates were washed under running tap water and dried at 50 °C for 2 h. SRB stain (0.1 mL) was added to each well and stored in the dark. After 1 h, the plates were washed with 1% acetic acid (0.1 mL × 4). The plates were dried again, and 10 mM tris buffer (0.1 mL) was added to each well to solubilise the dye. The plates were shaken gently for 1 h and the absorbance was measured at 510 nm. The growth inhibition of the test compounds was determined as a percentage of the optical density of the control group. Optical density was measured on an MRC UT-6100 Microplate 8-reading channel reader (Biocom Biotech, Pretoria, South Africa). A one-way ANOVA with Dunnett’s post hoc test was performed using GraphPad Prism version 5.00 for Windows, GraphPad Software, San Diego, CA, USA. Data were fitted to a non-linear regression of normalised response.

## 4. Conclusions

The single-crystal X-ray-determined structure of **5**, [Ir(ppy)_2_(FcCOCHCORc)], showed that both metallocenyl groups project towards the same side of the β-diketonato pseudo-aromatic plane, but the ruthenocenyl group is bent out of the plane by 28.17^o^ and the ferrocenyl group is only bent by 11.63°. This is attributed to the larger-sized ruthenocenyl group (Cp–Cp distance = 3.502 Å) compared to the ferrocenyl group (Cp-Cp distance = 3.422 Å) having to fit into limited available space.

An XPS study showed that the electron density on an atom expressed as Σχ_R_ influences the binding energy (BE) of N 1s, Ru 3p_3/2_ or 3d_5/2_, and Ir 4f_7/2_ photoelectrons differently than for Fe 2p_3/2_. BEs for the first mentioned elements in **1**–**6** decreased with increasing χ_R_ or Σχ_R_ whereas it usually decreases as demonstrated here and elsewhere for the first-row elements Mn [65], Cu [66], Co [67], Cr [68], and Fe [65]. DFT calculations to obtain orbital energies, although confirming trends, did not provide an explanation of why the obtained XPS BE—Σχ_R_ trends were opposite to the expected. Numerically, the DFT orbital energies have the expected negative signs but were found in much smaller energy ranges than BE values. For example, Ir 4f_7/2_ BEs were in the range of 61.4 < Ir 4f_7/2_ BE < 61.9 eV (i.e., 0.5 eV), but DFT orbital energies for the Ir 4f orbital only spanned the range −60.98 to −60.88 eV, that is, 0.1 eV. Plots of XPS BEs vs. DFT-calculated orbital energies had the required negative slopes, but because of these different numerical ranges, they were as large as −10 compared to the expected −1. We conclude that the main quantum level, n = 1, 2, 3…, and sub-quantum level (s, p, d, and f) all play a unique role in quantifying these XPS BE—Σχ_R_ trends, and they may differ from compound series to compound series. Several reasons exist why differences between XPS BE—Σχ_R_ trends and (DFT orbital energy calculations)—Σχ_R_ trends are so large. DFT calculations provide the energies of core molecular orbitals involved in the XPS excitation of electrons. Although these DFT orbital energies approximate the ionisation energies (IEs) observed in X-ray photoelectron spectroscopy (XPS), they are not exact XPS energies, as they either very poorly or do not account at all for post photoemission relaxation effects. In addition, XPS directly measures the IEs of individual core electrons, which are highly sensitive to the chemical and physical environment of the ionised atomic site. DFT calculates the energies of an entire orbital; for example, it calculated the orbital energies of each of the fourteen Ir 4f electrons residing in the t_1u_ and t_2u_ en a_2u_ orbitals (when referring to the ligand field splitting of f orbitals in an octahedral ligand field). From these, one must select the electrons that closely resemble the sought XPS electron energy and then average these values as an estimate of the specific electron’s BE. This is why we specified in the experimental section, for example, for Ir, that the average energy of the four lowest energy Ir 4f orbitals, (t_2u_ + a_2u_)_ave_, were related to the Ir 4f_7/2_ XPS BEs and why, in Figure 4, XPS BEs are labelled Ir 4f_7/2_ but the DFT-calculated Ir 4f energies are labelled “Ir 4f DFT-calculated average orbital energy”. DFT calculations on small molecules have modelled XPS binding energies accurately [89]. Due to the computational demands of using a large basis set for core-excited transition metal atoms of large molecules such as **1**–**6** studied here, XPS calculations are difficult or often even impractical. As for future research, a literature search of DFT orbital energy calculations related to XPS binding energies of, for example, the Ir 4f_7/2_ orbital, as well as XPS studies on even more common iridium salts, will show that it has basically not been addressed for small or large molecules such as **1**–**6**. The quest to understand the role of different final state energy relaxation effects in large molecules and to accurately calculate XPS binding energies with DFT methods is a topic of future research, especially by theoretical chemists, once computer power and DFT base sets for d and f electrons have been developed and/or refined much more.

The obtained relationships demonstrate that good electronic communication exists between molecular fragments in each complex. The observed X-ray-induced damage of **1**–**6** in the Fe, Ru, and Ir photoelectron lines with increased exposure time cautions that XPS studies should be conducted with a minimum exposure time to eliminate or minimise side effects of radiation-induced compound damage.

Finally, **1**–**6** do not exhibit antibacterial activity, but [Ir^III^(ppy)_2_(FcCOCHCOCH_3_)], **3**, is more cytotoxic than the free FcCOCH_2_COCH_3_ ligand. [Ir^III^(ppy)_2_(CH_3_COCHCOCH_3_)], **1**, is more cytotoxic than **3.** Both **1** and **3** are less cytotoxic than cisplatin.

## Figures and Tables

**Table 1:** Crystal data and structural refinement for **5**.

empirical formula	C_45_H_35_IrN_2_O_2_FeRu	absorption coeff. (mm^−1^)	4.408
molecular weight	983.4	θ range for data collection (deg)	2.190–26.371
crystal size (mm^3^)	0.488 × 0.331 × 0.162	index ranges	−19 ≤ h ≤ 19
temperature (K)	150.0(2)		−16 ≤ k ≤ 16
wavelength (Å)	0.71073		−22 ≤ l ≤ 22
crystal system	monoclinic	no. of reflections collected	100,881
space group	P 2_1_/n	no. of independent reflections	7500
unit cell dim. (Å; deg)	*a* = 15.850(3); *α* = 90	completeness to θ = 25.00°	98.9%
	*b* = 13.330(2); *β* = 101.390(6)	refinement method	full-matrix least squares on F^2^
	*c* = 17.904(3); *γ* = 90	data/restraints/parameters	7500/0/471
volume (Å^3^)	3708.3(11)	goodness of fit on *F*^2^	1.124
Z	4	final *R* indices [*I* > 2σ(I)]	R1 = 0.0371; wR2 = 0.0772
density (calc.) (g cm^−3^)	1.761	*R* indices (all data)	R1 = 0.0497; wR2 = 0.0707
*F*(000)	1926	largest diff. peak and hole (e Å^−3^)	1.751 and −0.824

The asymmetric unit cell contains positional disorder of the Fe and Ru atoms which occupy each other’s positions about 50% of the time.

**Table 3:** Σχ_R_ values, binding energies (BEs) of Ru 3d_5/2_ and 3p_3/2_ and Fe 2p_3/2_ compounds, decomposition product photoelectron lines, DFT-calculated orbital energies (eV), and I_ratio_ values for **1**–**6**.

	Σχ_R_	Binding Energies for Ru (eV)				DFT Orb. En. (eV)			I_ratio_ ^a,b^	BE of Fe		I_ratio_^a,b^
		3d_5/2_	3d_5/2 dec_	3p_3/2_	3p_3/2 dec_	Ru (3d-t_2g_)_ave_	Ru (3p-π)_ave_	Fe (2p-π)_ave_	Ru 3p_3/2_	2p_3/2_	2p_3/2 dec_	Fe 2p_3/2_
**1**	4.68											
**2**	4.33	281.1	279.5	461.3	457.2	−274.05	−447.42		0.24 (0.889) ^b^			
**3**	4.21							−695.77		708.0	705.4	0.25 (0.086) ^b^
**4**	3.98	281.2	279.5	462.1	458.5	−274.17	−447.55		0.31 (0.771) ^b^			
**5**	3.86	281.3	279.4	461.5	457.9	−274.13	−447.50	−695.91	0.19 (1.013) ^b^	707.8	705.9	0.25 (0.073) ^b^
**6**	3.74							−695.87		707.3	705.1	0.14 (0.050) ^b^

^a^ I_ratio_ = ratio between the intensities of the decomposition product and main compound photoelectron line intensities. ^b^ Values in brackets are the electrochemically determined oxidation potentials, E_pa_, of Rc in volt or the reversible formal reduction potential, E°′, of the Fc/Fc^+^ redox couple in volt as per [1].

**Table 4:** IC_50_ values of **1**, **3**, and cisplatin against HeLa cells expressed as mean drug concentration ± mean error, from the mean of three experiments.

Compound	IC_50_ (μM)
Cisplatin	1.1 ± 0.1
[Ir^III^(ppy)_2_(CH_3_COCHCOCH_3_)], **1**	25.1 ± 0.2
[Ir^III^(ppy)_2_(FcCOCHCOCH_3_)], **3**	37.8 ± 0.2

## Data Availability

CCDC 2307998 contains the supplementary crystallographic data for this paper. These data can be obtained free of charge via www.ccdc.cam.ac.uk/data_request/cif, by emailing data_request@ccdc.cam.ac.uk, or by contacting The Cambridge Crystallographic Data Centre, 12 Union Road, Cambridge, CB2 1EZ, UK, Fax: +44-1223-336033.

## Supplementary Materials

The following supporting information can be downloaded at: https://www.mdpi.com/article/10.3390/molecules29225383/s1, Detailed XPS spectra of the Fe 2p region of FcCOCH_2_CORc, the N 1s region of **1**–**6**, and Ru 3d regions of **1**–**6**. Figures highlighting XPS and DFT relationships of **1**–**6**. Tables providing crystallographic information and optimised coordinates from DFT calculations of free β-diketones and **1**–**6**.
