# Supplementary material for: Unexpected XPS Binding Energy Observations Further Highlighted by DFT Calculations of Ruthenocene-Containing [IrIII(ppy)2(RCOCHCORc)] Complexes: Cytotoxicity and Crystal Structure of [Ir(ppy)2(FcCOCHCORc)]
Source: Molecules. 2024 Nov 15;29(22):5383. doi: 10.3390/molecules29225383 (PMC11597539; doi:10.3390/molecules29225383)
Supplement: Supplementary file 1 [file molecules-29-05383-s001.zip › molecules-3260138-supplementary.pdf]

Supplementary Information for

Unexpected XPS Binding Energy Observations Further Highlighted by DFT Calculations of Ruthenocene-Containing $[Ir^{III}(ppy)_2(RCOCHCORc)]$ Complexes. Cytotoxicity and Crystal Structure of $[Ir(ppy)_2(FcCOCHCORc)]$

Blenerhassitt E. Buitendach,[a] Elizabeth Erasmus,[a] Eleanor Fourie,[a] Frederick P. Malan,[b] Jeanet Conradie,[a] J. W. (Hans) Niemantsverdriet,[c] and Jannie C. Swarts[a]

A. Figure S1. XPS spectrum of $FcCOCH_2CORc$` **p 2**

B. Figure S2. XPS spectra of the N 1s area of 1-6 **p 2**

C. XPS spectra and relationships of the Ru 3p and 3d area of 2, 4 and 5 **p 3**
- **Figure S3.** XPS spectrums of the Ru 3d area of 2, 4 and 5 p 3
- **Figure S4**. Plots of Ru $3p_{3/2}$ or Ru$3d_{5/2}$ binding energies against Ru peak anodic potentials (E_{pa}), or $\Sigma\chi_R$, or DFT-calculated orbital energies of **2**, **4** and **5** failed to give clear relationships p 3

D. Figure S5. XPS and DFT relationships of the Fe 2p area of 3, 5 and 6 **p 4**

E. Crystallographic Information of 5 **p 5**
- **Table 1.** Bond lengths for **5** p 5
- **Table 2.** Bond angles for **5** p 6
- **Table 3.** Torsion angles for **5** p 10
- **Table 4.** Atomic occupancy for **5** p 12

F. DFT calculations: Optimised Cartesian coordinates for compounds, (Å) **p13**
1. $RcCOCH_2COFc$ p 13
2. $RcCOCH_2CORc$ p 14
3. $RcCOCH_2COCH_3$ p 15
4. $[Ir(ppy)_2(CH_3COCHCOCH_3)]$, **1** p 15
5. $[Ir(ppy)_2(CH_3COCHCORc)]$, **2** p 16
6. $[Ir(ppy)_2(CH_3COCHCOFc)]$, **3** p 18
7. $[Ir(ppy)_2(RcCOCHCORc)]$, **4** p 19
8. $[Ir(ppy)_2(RcCOCHCOFc)]$, **5** p 21
9. $[Ir(ppy)_2(FcCOCHCOFc)]$, **6** p 23

A. XPS spectrum of $FcCOCH_2CORc$

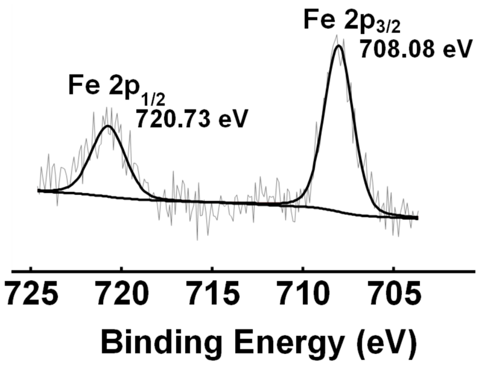

Figure S1. XPS spectrum showing the Fe 2p binding energy envelope of $FcCOCH_2CORc$

B. XPS spectra of the N 1s area of 1-6.

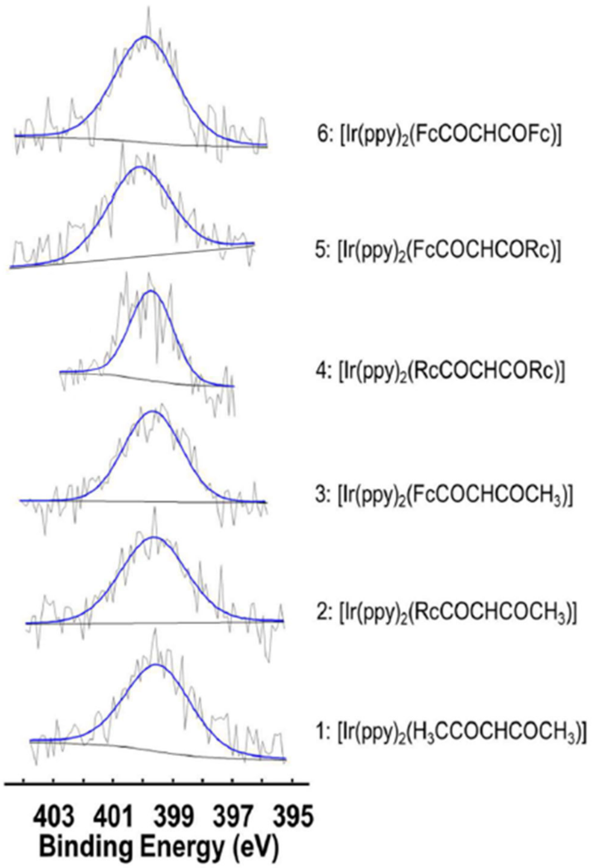

Figure S2. XPS spectra showing the N 1s area of **1-6**.

C. XPS spectra and relationships of the Ru 3p and 3d area of 2, 4 and 5

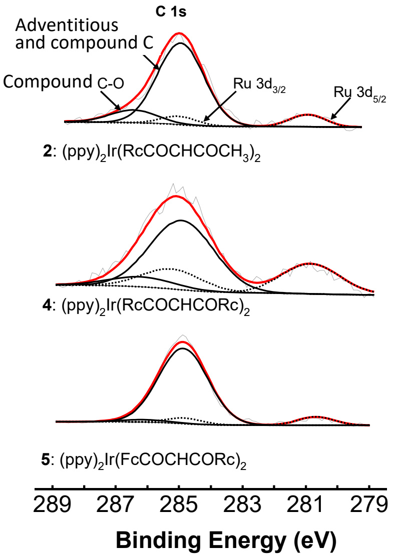

Figure S3. XPS spectra showing the Ru 3d/C 1s area of **2, 4** and **5**.

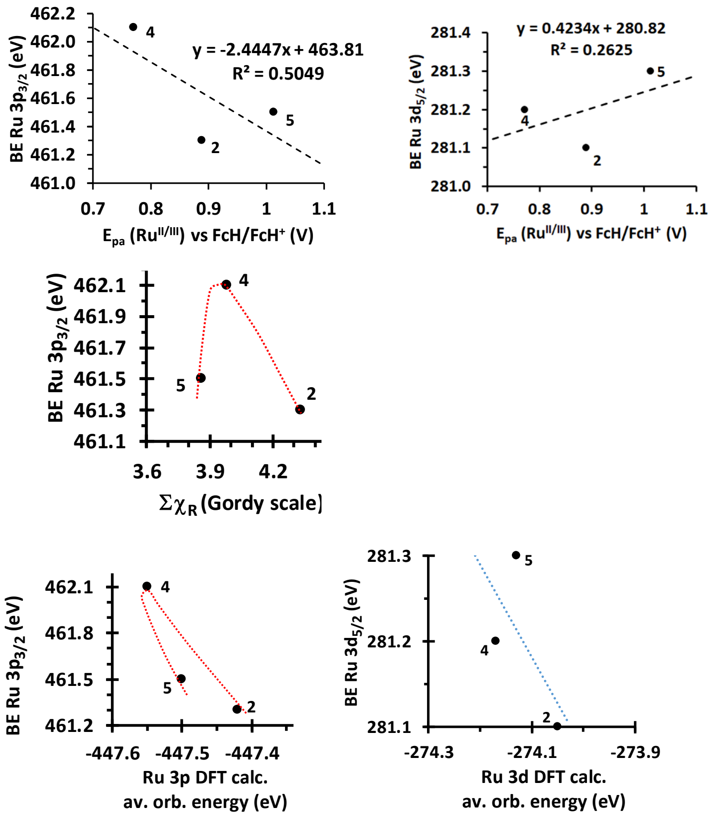

Figure S4. Plots of Ru $3p_{3/2}$ or Ru$3d_{5/2}$ binding energies against Ru peak anodic potentials (E_{pa}, potentials from reference 1), or $\Sigma\chi_R$, or DFT-calculated orbital energies of **2**, **4** and **5** failed to give clear relationships.

D. XPS and DFT relationships of the Fe 2p area of 3, 5 and 6

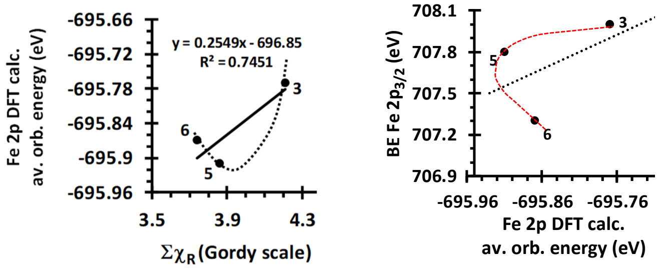

Figure S5. XPS and DFT relationships of the Fe 2p area of **3**, **5** and **6**

E. Crystallographic Information of 5

Table S1. Bond Lengths for 5.

Atom	Atom	Length/Å	Atom	Atom	Length/Å
Ir1	O1	2.137(3)	N1	C5	1.363(7)
Ir1	O2	2.163(3)	N2	C12	1.345(7)
Ir1	N1	2.032(4)	N2	C16	1.366(7)
Ir1	N2	2.037(4)	C1	C2	1.366(8)
Ir1	C11	1.992(5)	C2	C3	1.379(9)
Ir1	C22	2.000(5)	C3	C4	1.369(9)
Ru1	C36	2.131(5)	C4	C5	1.399(8)
Ru1	C37	2.138(5)	C5	C6	1.463(8)
Ru1	C38	2.129(6)	C6	C7	1.388(7)
Ru1	C39	2.132(6)	C6	C11	1.409(7)
Ru1	C40	2.126(6)	C7	C8	1.383(8)
Ru1	C41	2.131(7)	C8	C9	1.384(8)
Ru1	C42	2.121(7)	C9	C10	1.391(7)
Ru1	C43	2.125(7)	C10	C11	1.394(7)
Ru1	C44	2.138(6)	C12	C13	1.375(8)
Ru1	C45	2.139(7)	C13	C14	1.397(10)
Fe1	C26	2.121(5)	C14	C15	1.376(9)
Fe1	C27	2.102(6)	C15	C16	1.392(8)
Fe1	C28	2.086(7)	C16	C17	1.447(8)
Fe1	C29	2.097(6)	C17	C18	1.416(7)
Fe1	C30	2.106(6)	C17	C22	1.419(7)
Fe1	C31	2.095(8)	C18	C19	1.369(9)
Fe1	C32	2.080(8)	C19	C20	1.387(9)
Fe1	C33	2.079(8)	C20	C21	1.393(7)
Fe1	C34	2.092(7)	C21	C22	1.390(8)
Fe1	C35	2.086(8)	C23	C24	1.402(7)
Fe2	C36	2.131(5)	C23	C26	1.471(7)
Fe2	C37	2.138(5)	C24	C25	1.400(7)
Fe2	C38	2.129(6)	C25	C36	1.482(7)
Fe2	C39	2.132(6)	C26	C27	1.435(8)
Fe2	C40	2.126(6)	C26	C30	1.442(7)
Fe2	C41	2.131(7)	C27	C28	1.416(8)
Fe2	C42	2.121(7)	C28	C29	1.415(9)
Fe2	C43	2.125(7)	C29	C30	1.420(8)
Fe2	C44	2.138(6)	C31	C32	1.434(12)
Fe2	C45	2.139(7)	C31	C35	1.405(12)
Ru2	C26	2.121(5)	C32	C33	1.398(13)
Ru2	C27	2.102(6)	C33	C34	1.424(12)
Ru2	C28	2.086(7)	C34	C35	1.408(11)
Ru2	C29	2.097(6)	C36	C37	1.430(8)
Ru2	C30	2.106(6)	C36	C40	1.437(8)

Table S1. Bond Lengths for 5.

Atom	Atom	Length/Å	Atom	Atom	Length/Å
Ru2	C31	2.095(8)	C37	C38	1.425(8)
Ru2	C32	2.080(8)	C38	C39	1.421(9)
Ru2	C33	2.079(8)	C39	C40	1.422(8)
Ru2	C34	2.092(7)	C41	C42	1.425(11)
Ru2	C35	2.086(8)	C41	C45	1.417(10)
O1	C23	1.271(6)	C42	C43	1.421(10)
O2	C25	1.271(6)	C43	C44	1.414(10)
N1	C1	1.351(7)	C44	C45	1.416(11)

Table S2. Bond Angles for 5.

Atom	Atom	Atom	Angle/°	Atom	Atom	Atom	Angle/°
O1	Ir1	O2	89.27(13)	C33	Ru2	C28	124.1(3)
N1	Ir1	O1	88.24(15)	C33	Ru2	C29	107.5(3)
N1	Ir1	O2	92.94(16)	C33	Ru2	C30	121.5(3)
N1	Ir1	N2	174.12(17)	C33	Ru2	C31	67.5(4)
N2	Ir1	O1	95.35(16)	C33	Ru2	C32	39.3(4)
N2	Ir1	O2	91.77(15)	C33	Ru2	C34	39.9(3)
C11	Ir1	O1	89.44(16)	C33	Ru2	C35	66.5(3)
C11	Ir1	O2	173.75(17)	C34	Ru2	C26	124.1(3)
C11	Ir1	N1	80.91(19)	C34	Ru2	C27	158.5(3)
C11	Ir1	N2	94.44(18)	C34	Ru2	C29	125.9(3)
C11	Ir1	C22	89.42(19)	C34	Ru2	C30	110.4(3)
C22	Ir1	O1	175.72(18)	C34	Ru2	C31	66.8(4)
C22	Ir1	O2	92.30(16)	C35	Ru2	C26	112.2(3)
C22	Ir1	N1	95.65(19)	C35	Ru2	C27	124.5(3)
C22	Ir1	N2	80.6(2)	C35	Ru2	C28	157.1(3)
C36	Ru1	C37	39.1(2)	C35	Ru2	C29	163.0(3)
C36	Ru1	C39	65.7(2)	C35	Ru2	C30	128.6(3)
C36	Ru1	C41	160.6(3)	C35	Ru2	C31	39.3(3)
C36	Ru1	C44	110.4(2)	C35	Ru2	C34	39.4(3)
C36	Ru1	C45	125.8(3)	C23	O1	Ir1	124.1(3)
C37	Ru1	C45	111.2(3)	C25	O2	Ir1	123.6(3)
C38	Ru1	C36	65.8(2)	C1	N1	Ir1	125.1(4)
C38	Ru1	C37	39.0(2)	C1	N1	C5	119.5(5)
C38	Ru1	C39	39.0(2)	C5	N1	Ir1	115.4(3)
C38	Ru1	C41	111.0(3)	C12	N2	Ir1	123.8(4)
C38	Ru1	C44	158.9(3)	C12	N2	C16	120.1(5)
C38	Ru1	C45	125.1(3)	C16	N2	Ir1	116.0(4)
C39	Ru1	C37	65.3(2)	N1	C1	C2	122.2(6)
C39	Ru1	C44	160.7(3)	C1	C2	C3	119.3(5)
C39	Ru1	C45	159.1(3)	C4	C3	C2	119.2(6)

Table S2. Bond Angles for 5.

Atom	Atom	Atom	Angle/°	Atom	Atom	Atom	Angle/°
C40	Ru1	C36	39.5(2)	C3	C4	C5	120.5(6)
C40	Ru1	C37	65.6(2)	N1	C5	C4	119.4(5)
C40	Ru1	C38	65.6(2)	N1	C5	C6	114.3(5)
C40	Ru1	C39	39.0(2)	C4	C5	C6	126.4(5)
C40	Ru1	C41	158.8(3)	C7	C6	C5	124.1(5)
C40	Ru1	C44	125.8(3)	C7	C6	C11	121.3(5)
C40	Ru1	C45	160.7(3)	C11	C6	C5	114.6(5)
C41	Ru1	C37	126.1(3)	C8	C7	C6	120.3(5)
C41	Ru1	C39	124.9(3)	C7	C8	C9	119.1(5)
C41	Ru1	C44	65.1(3)	C8	C9	C10	120.9(5)
C41	Ru1	C45	38.8(3)	C9	C10	C11	120.9(5)
C42	Ru1	C36	158.5(3)	C6	C11	Ir1	114.6(4)
C42	Ru1	C37	161.0(3)	C10	C11	Ir1	128.0(4)
C42	Ru1	C38	126.1(3)	C10	C11	C6	117.3(5)
C42	Ru1	C39	110.8(3)	N2	C12	C13	121.4(6)
C42	Ru1	C40	124.2(3)	C12	C13	C14	119.7(6)
C42	Ru1	C41	39.2(3)	C15	C14	C13	118.5(6)
C42	Ru1	C43	39.1(3)	C14	C15	C16	120.4(6)
C42	Ru1	C44	65.3(3)	N2	C16	C15	119.9(5)
C42	Ru1	C45	65.2(3)	N2	C16	C17	113.7(4)
C43	Ru1	C36	124.1(3)	C15	C16	C17	126.3(5)
C43	Ru1	C37	158.5(3)	C18	C17	C16	123.5(5)
C43	Ru1	C38	161.0(3)	C18	C17	C22	120.7(5)
C43	Ru1	C39	126.1(3)	C22	C17	C16	115.7(5)
C43	Ru1	C40	110.5(3)	C19	C18	C17	119.6(5)
C43	Ru1	C41	65.4(3)	C18	C19	C20	120.6(5)
C43	Ru1	C44	38.8(3)	C19	C20	C21	119.9(6)
C43	Ru1	C45	64.9(3)	C22	C21	C20	121.9(5)
C44	Ru1	C37	124.7(2)	C17	C22	Ir1	113.9(4)
C44	Ru1	C45	38.7(3)	C21	C22	Ir1	128.8(4)
C27	Fe1	C26	39.7(2)	C21	C22	C17	117.3(5)
C27	Fe1	C30	66.6(2)	O1	C23	C24	126.9(5)
C28	Fe1	C26	66.5(2)	O1	C23	C26	115.0(4)
C28	Fe1	C27	39.5(2)	C24	C23	C26	118.2(5)
C28	Fe1	C29	39.5(3)	C25	C24	C23	128.4(5)
C28	Fe1	C30	66.4(3)	O2	C25	C24	127.1(5)
C28	Fe1	C31	121.7(3)	O2	C25	C36	115.2(5)
C28	Fe1	C34	160.9(3)	C24	C25	C36	117.6(5)
C29	Fe1	C26	66.6(2)	C23	C26	Fe1	125.1(4)
C29	Fe1	C27	66.5(2)	C23	C26	Ru2	125.1(4)
C29	Fe1	C30	39.5(2)	C27	C26	Fe1	69.4(3)
C30	Fe1	C26	39.9(2)	C27	C26	Ru2	69.4(3)
C31	Fe1	C26	127.2(3)	C27	C26	C23	128.3(5)

Table S2. Bond Angles for 5.

Atom	Atom	Atom	Angle/°	Atom	Atom	Atom	Angle/°
C31	Fe1	C27	109.7(3)	C27	C26	C30	106.9(5)
C31	Fe1	C29	155.3(3)	C30	C26	Fe1	69.5(3)
C31	Fe1	C30	163.8(3)	C30	C26	Ru2	69.5(3)
C32	Fe1	C26	163.1(4)	C30	C26	C23	124.7(5)
C32	Fe1	C27	126.2(4)	C26	C27	Fe1	70.9(3)
C32	Fe1	C28	108.5(3)	C26	C27	Ru2	70.9(3)
C32	Fe1	C29	120.7(3)	C28	C27	Fe1	69.7(4)
C32	Fe1	C30	154.9(3)	C28	C27	Ru2	69.7(4)
C32	Fe1	C31	40.2(3)	C28	C27	C26	108.1(5)
C32	Fe1	C34	65.9(4)	C27	C28	Fe1	70.8(4)
C32	Fe1	C35	65.6(3)	C27	C28	Ru2	70.8(4)
C33	Fe1	C26	157.1(3)	C29	C28	Fe1	70.6(4)
C33	Fe1	C27	160.5(3)	C29	C28	Ru2	70.6(4)
C33	Fe1	C28	124.1(3)	C29	C28	C27	108.8(6)
C33	Fe1	C29	107.5(3)	C28	C29	Fe1	69.8(4)
C33	Fe1	C30	121.5(3)	C28	C29	Ru2	69.8(4)
C33	Fe1	C31	67.5(4)	C28	C29	C30	108.1(5)
C33	Fe1	C32	39.3(4)	C30	C29	Fe1	70.6(3)
C33	Fe1	C34	39.9(3)	C30	C29	Ru2	70.6(3)
C33	Fe1	C35	66.5(3)	C26	C30	Fe1	70.6(3)
C34	Fe1	C26	124.1(3)	C26	C30	Ru2	70.6(3)
C34	Fe1	C27	158.5(3)	C29	C30	Fe1	69.9(4)
C34	Fe1	C29	125.9(3)	C29	C30	Ru2	69.9(4)
C34	Fe1	C30	110.4(3)	C29	C30	C26	108.1(5)
C34	Fe1	C31	66.8(4)	C32	C31	Fe1	69.4(5)
C35	Fe1	C26	112.2(3)	C32	C31	Ru2	69.4(5)
C35	Fe1	C27	124.5(3)	C35	C31	Fe1	70.0(5)
C35	Fe1	C28	157.1(3)	C35	C31	Ru2	70.0(5)
C35	Fe1	C29	163.0(3)	C35	C31	C32	105.4(8)
C35	Fe1	C30	128.6(3)	C31	C32	Fe1	70.5(5)
C35	Fe1	C31	39.3(3)	C31	C32	Ru2	70.5(5)
C35	Fe1	C34	39.4(3)	C33	C32	Fe1	70.3(5)
C36	Fe2	C37	39.1(2)	C33	C32	Ru2	70.3(5)
C36	Fe2	C39	65.7(2)	C33	C32	C31	110.0(8)
C36	Fe2	C41	160.6(3)	C32	C33	Fe1	70.4(5)
C36	Fe2	C44	110.4(2)	C32	C33	Ru2	70.4(5)
C36	Fe2	C45	125.8(3)	C32	C33	C34	107.1(8)
C37	Fe2	C45	111.2(3)	C34	C33	Fe1	70.5(4)
C38	Fe2	C36	65.8(2)	C34	C33	Ru2	70.5(4)
C38	Fe2	C37	39.0(2)	C33	C34	Fe1	69.6(5)
C38	Fe2	C39	39.0(2)	C33	C34	Ru2	69.6(5)
C38	Fe2	C41	111.0(3)	C35	C34	Fe1	70.1(5)
C38	Fe2	C44	158.9(3)	C35	C34	Ru2	70.1(5)

Table S2. Bond Angles for 5.

Atom	Atom	Atom	Angle/°	Atom	Atom	Atom	Angle/°
C38	Fe2	C45	125.1(3)	C35	C34	C33	107.5(8)
C39	Fe2	C37	65.3(2)	C31	C35	Fe1	70.7(5)
C39	Fe2	C44	160.7(3)	C31	C35	Ru2	70.7(5)
C39	Fe2	C45	159.1(3)	C31	C35	C34	110.1(7)
C40	Fe2	C36	39.5(2)	C34	C35	Fe1	70.5(4)
C40	Fe2	C37	65.6(2)	C34	C35	Ru2	70.5(4)
C40	Fe2	C38	65.6(2)	C25	C36	Ru1	122.0(3)
C40	Fe2	C39	39.0(2)	C25	C36	Fe2	122.0(3)
C40	Fe2	C41	158.8(3)	C37	C36	Ru1	70.7(3)
C40	Fe2	C44	125.8(3)	C37	C36	Fe2	70.7(3)
C40	Fe2	C45	160.7(3)	C37	C36	C25	124.9(5)
C41	Fe2	C37	126.1(3)	C37	C36	C40	107.3(5)
C41	Fe2	C39	124.9(3)	C40	C36	Ru1	70.1(3)
C41	Fe2	C44	65.1(3)	C40	C36	Fe2	70.1(3)
C41	Fe2	C45	38.8(3)	C40	C36	C25	127.7(5)
C42	Fe2	C36	158.5(3)	C36	C37	Ru1	70.2(3)
C42	Fe2	C37	161.0(3)	C36	C37	Fe2	70.2(3)
C42	Fe2	C38	126.1(3)	C38	C37	Ru1	70.2(3)
C42	Fe2	C39	110.8(3)	C38	C37	Fe2	70.2(3)
C42	Fe2	C40	124.2(3)	C38	C37	C36	108.3(5)
C42	Fe2	C41	39.2(3)	C37	C38	Ru1	70.8(3)
C42	Fe2	C43	39.1(3)	C37	C38	Fe2	70.8(3)
C42	Fe2	C44	65.3(3)	C39	C38	Ru1	70.6(3)
C42	Fe2	C45	65.2(3)	C39	C38	Fe2	70.6(3)
C43	Fe2	C36	124.1(3)	C39	C38	C37	108.1(5)
C43	Fe2	C37	158.5(3)	C38	C39	Ru1	70.4(3)
C43	Fe2	C38	161.0(3)	C38	C39	Fe2	70.4(3)
C43	Fe2	C39	126.1(3)	C38	C39	C40	108.3(5)
C43	Fe2	C40	110.5(3)	C40	C39	Ru1	70.3(3)
C43	Fe2	C41	65.4(3)	C40	C39	Fe2	70.3(3)
C43	Fe2	C44	38.8(3)	C36	C40	Ru1	70.4(3)
C43	Fe2	C45	64.9(3)	C36	C40	Fe2	70.4(3)
C44	Fe2	C37	124.7(2)	C39	C40	Ru1	70.7(3)
C44	Fe2	C45	38.7(3)	C39	C40	Fe2	70.7(3)
C27	Ru2	C26	39.7(2)	C39	C40	C36	108.0(5)
C27	Ru2	C30	66.6(2)	C42	C41	Ru1	70.0(4)
C28	Ru2	C26	66.5(2)	C42	C41	Fe2	70.0(4)
C28	Ru2	C27	39.5(2)	C45	C41	Ru1	70.9(4)
C28	Ru2	C29	39.5(3)	C45	C41	Fe2	70.9(4)
C28	Ru2	C30	66.4(3)	C45	C41	C42	107.7(7)
C28	Ru2	C31	121.7(3)	C41	C42	Ru1	70.8(4)
C28	Ru2	C34	160.9(3)	C41	C42	Fe2	70.8(4)
C29	Ru2	C26	66.6(2)	C43	C42	Ru1	70.6(4)

Table S2. Bond Angles for 5.

Atom	Atom	Atom	Angle/°	Atom	Atom	Atom	Angle/°
C29	Ru2	C27	66.5(2)	C43	C42	Fe2	70.6(4)
C29	Ru2	C30	39.5(2)	C43	C42	C41	107.7(7)
C30	Ru2	C26	39.9(2)	C42	C43	Ru1	70.3(4)
C31	Ru2	C26	127.2(3)	C42	C43	Fe2	70.3(4)
C31	Ru2	C27	109.7(3)	C44	C43	Ru1	71.1(4)
C31	Ru2	C29	155.3(3)	C44	C43	Fe2	71.1(4)
C31	Ru2	C30	163.8(3)	C44	C43	C42	108.2(7)
C32	Ru2	C26	163.1(4)	C43	C44	Ru1	70.2(4)
C32	Ru2	C27	126.2(4)	C43	C44	Fe2	70.2(4)
C32	Ru2	C28	108.5(3)	C43	C44	C45	108.0(7)
C32	Ru2	C29	120.7(3)	C45	C44	Ru1	70.7(4)
C32	Ru2	C30	154.9(3)	C45	C44	Fe2	70.7(4)
C32	Ru2	C31	40.2(3)	C41	C45	Ru1	70.3(4)
C32	Ru2	C34	65.9(4)	C41	C45	Fe2	70.3(4)
C32	Ru2	C35	65.6(3)	C44	C45	Ru1	70.6(4)
C33	Ru2	C26	157.1(3)	C44	C45	Fe2	70.6(4)
C33	Ru2	C27	160.5(3)	C44	C45	C41	108.4(7)

Table S3. Torsion Angles for 5.

A	B	C	D	Angle/°	A	B	C	D	Angle/°
Ir1	O1	C23	C24	-10.0(7)	C16	C17	C18	C19	174.4(5)
Ir1	O1	C23	C26	170.4(3)	C16	C17	C22	Ir1	4.5(5)
Ir1	O2	C25	C24	1.3(7)	C16	C17	C22	C21	-174.3(4)
Ir1	O2	C25	C36	-175.7(3)	C17	C18	C19	C20	0.4(8)
Ir1	N1	C1	C2	-179.9(4)	C18	C17	C22	Ir1	-178.8(4)
Ir1	N1	C5	C4	-179.5(4)	C18	C17	C22	C21	2.3(7)
Ir1	N1	C5	C6	1.0(6)	C18	C19	C20	C21	0.8(9)
Ir1	N2	C12	C13	173.8(4)	C19	C20	C21	C22	-0.4(8)
Ir1	N2	C16	C15	-175.2(4)	C20	C21	C22	Ir1	-179.7(4)
Ir1	N2	C16	C17	1.2(5)	C20	C21	C22	C17	-1.1(7)
Ru1	C36	C37	C38	60.1(4)	C22	C17	C18	C19	-2.0(8)
Ru1	C36	C40	C39	-61.0(4)	C23	C24	C25	O2	-1.0(9)
Ru1	C37	C38	C39	61.1(4)	C23	C24	C25	C36	176.0(5)
Ru1	C38	C39	C40	60.3(4)	C23	C26	C27	Fe1	119.1(6)
Ru1	C39	C40	C36	60.8(4)	C23	C26	C27	Ru2	119.1(6)
Ru1	C41	C42	C43	-61.3(5)	C23	C26	C27	C28	179.1(6)
Ru1	C41	C45	C44	60.7(5)	C23	C26	C30	Fe1	-119.2(5)
Ru1	C42	C43	C44	-61.3(5)	C23	C26	C30	Ru2	-119.2(5)
Ru1	C43	C44	C45	-60.9(5)	C23	C26	C30	C29	-179.3(5)
Ru1	C44	C45	C41	-60.5(5)	C24	C23	C26	Fe1	99.6(5)
Fe1	C26	C27	C28	60.0(5)	C24	C23	C26	Ru2	99.6(5)

Table S3. Torsion Angles for 5.

A	B	C	D	Angle/°	A	B	C	D	Angle/°
Fe1	C26	C30	C29	-60.1(4)	C24	C23	C26	C27	9.1(9)
Fe1	C27	C28	C29	60.7(5)	C24	C23	C26	C30	-172.4(5)
Fe1	C28	C29	C30	60.5(4)	C24	C25	C36	Ru1	-61.0(6)
Fe1	C29	C30	C26	60.6(4)	C24	C25	C36	Fe2	-61.0(6)
Fe1	C31	C32	C33	-59.4(6)	C24	C25	C36	C37	-148.8(5)
Fe1	C31	C35	C34	59.7(6)	C24	C25	C36	C40	27.6(8)
Fe1	C32	C33	C34	-61.3(6)	C25	C36	C37	Ru1	116.2(5)
Fe1	C33	C34	C35	-60.1(6)	C25	C36	C37	Fe2	116.2(5)
Fe1	C34	C35	C31	-59.8(6)	C25	C36	C37	C38	176.2(5)
Fe2	C36	C37	C38	60.1(4)	C25	C36	C40	Ru1	-115.7(5)
Fe2	C36	C40	C39	-61.0(4)	C25	C36	C40	Fe2	-115.7(5)
Fe2	C37	C38	C39	61.1(4)	C25	C36	C40	C39	-176.7(5)
Fe2	C38	C39	C40	60.3(4)	C26	C23	C24	C25	-174.4(5)
Fe2	C39	C40	C36	60.8(4)	C26	C27	C28	Fe1	-60.7(4)
Fe2	C41	C42	C43	-61.3(5)	C26	C27	C28	Ru2	-60.7(4)
Fe2	C41	C45	C44	60.7(5)	C26	C27	C28	C29	0.0(8)
Fe2	C42	C43	C44	-61.3(5)	C27	C26	C30	Fe1	59.6(4)
Fe2	C43	C44	C45	-60.9(5)	C27	C26	C30	Ru2	59.6(4)
Fe2	C44	C45	C41	-60.5(5)	C27	C26	C30	C29	-0.5(7)
Ru2	C26	C27	C28	60.0(5)	C27	C28	C29	Fe1	-60.8(5)
Ru2	C26	C30	C29	-60.1(4)	C27	C28	C29	Ru2	-60.8(5)
Ru2	C27	C28	C29	60.7(5)	C27	C28	C29	C30	-0.3(8)
Ru2	C28	C29	C30	60.5(4)	C28	C29	C30	Fe1	-60.0(5)
Ru2	C29	C30	C26	60.6(4)	C28	C29	C30	Ru2	-60.0(5)
Ru2	C31	C32	C33	-59.4(6)	C28	C29	C30	C26	0.5(7)
Ru2	C31	C35	C34	59.7(6)	C30	C26	C27	Fe1	-59.6(4)
Ru2	C32	C33	C34	-61.3(6)	C30	C26	C27	Ru2	-59.6(4)
Ru2	C33	C34	C35	-60.1(6)	C30	C26	C27	C28	0.3(7)
Ru2	C34	C35	C31	-59.8(6)	C31	C32	C33	Fe1	59.5(6)
O1	C23	C24	C25	6.1(9)	C31	C32	C33	Ru2	59.5(6)
O1	C23	C26	Fe1	-80.8(6)	C31	C32	C33	C34	-1.8(10)
O1	C23	C26	Ru2	-80.8(6)	C32	C31	C35	Fe1	-60.7(6)
O1	C23	C26	C27	-171.3(6)	C32	C31	C35	Ru2	-60.7(6)
O1	C23	C26	C30	7.2(8)	C32	C31	C35	C34	-1.0(10)
O2	C25	C36	Ru1	116.3(4)	C32	C33	C34	Fe1	61.2(6)
O2	C25	C36	Fe2	116.3(4)	C32	C33	C34	Ru2	61.2(6)
O2	C25	C36	C37	28.5(7)	C32	C33	C34	C35	1.2(9)
O2	C25	C36	C40	-155.0(5)	C33	C34	C35	Fe1	59.8(6)
N1	C1	C2	C3	-0.5(8)	C33	C34	C35	Ru2	59.8(6)
N1	C5	C6	C7	-179.5(5)	C33	C34	C35	C31	-0.1(10)
N1	C5	C6	C11	2.7(7)	C35	C31	C32	Fe1	61.2(6)
N2	C12	C13	C14	1.3(9)	C35	C31	C32	Ru2	61.2(6)
N2	C16	C17	C18	179.7(5)	C35	C31	C32	C33	1.8(10)

Table S3. Torsion Angles for 5.

A	B	C	D	Angle/°	A	B	C	D	Angle/°
N2	C16	C17	C22	-3.7(6)	C36	C37	C38	Ru1	-60.1(4)
C1	N1	C5	C4	0.2(7)	C36	C37	C38	Fe2	-60.1(4)
C1	N1	C5	C6	-179.3(5)	C36	C37	C38	C39	1.1(6)
C1	C2	C3	C4	-0.3(9)	C37	C36	C40	Ru1	61.3(4)
C2	C3	C4	C5	1.0(9)	C37	C36	C40	Fe2	61.3(4)
C3	C4	C5	N1	-0.9(8)	C37	C36	C40	C39	0.2(6)
C3	C4	C5	C6	178.5(5)	C37	C38	C39	Ru1	-61.3(4)
C4	C5	C6	C7	1.0(9)	C37	C38	C39	Fe2	-61.3(4)
C4	C5	C6	C11	-176.8(5)	C37	C38	C39	C40	-0.9(7)
C5	N1	C1	C2	0.5(8)	C38	C39	C40	Ru1	-60.4(4)
C5	C6	C7	C8	-176.0(5)	C38	C39	C40	Fe2	-60.4(4)
C5	C6	C11	Ir1	-5.2(6)	C38	C39	C40	C36	0.4(7)
C5	C6	C11	C10	173.5(5)	C40	C36	C37	Ru1	-60.9(4)
C6	C7	C8	C9	2.3(8)	C40	C36	C37	Fe2	-60.9(4)
C7	C6	C11	Ir1	177.0(4)	C40	C36	C37	C38	-0.8(6)
C7	C6	C11	C10	-4.3(7)	C41	C42	C43	Ru1	61.4(5)
C7	C8	C9	C10	-3.4(8)	C41	C42	C43	Fe2	61.4(5)
C8	C9	C10	C11	0.6(8)	C41	C42	C43	C44	0.1(8)
C9	C10	C11	Ir1	-178.3(4)	C42	C41	C45	Ru1	-60.7(5)
C9	C10	C11	C6	3.2(7)	C42	C41	C45	Fe2	-60.7(5)
C11	C6	C7	C8	1.6(8)	C42	C41	C45	C44	0.0(8)
C12	N2	C16	C15	0.1(7)	C42	C43	C44	Ru1	60.8(5)
C12	N2	C16	C17	176.4(4)	C42	C43	C44	Fe2	60.8(5)
C12	C13	C14	C15	-0.5(9)	C42	C43	C44	C45	-0.1(8)
C13	C14	C15	C16	-0.5(9)	C43	C44	C45	Ru1	60.6(5)
C14	C15	C16	N2	0.7(8)	C43	C44	C45	Fe2	60.6(5)
C14	C15	C16	C17	-175.2(5)	C43	C44	C45	C41	0.1(8)
C15	C16	C17	C18	-4.2(8)	C45	C41	C42	Ru1	61.2(5)
C15	C16	C17	C22	172.4(5)	C45	C41	C42	Fe2	61.2(5)
C16	N2	C12	C13	-1.0(8)	C45	C41	C42	C43	0.0(8)

Table S4. Atomic Occupancy for 5.

Atom	*Occupancy*	Atom	*Occupancy*
Ru1	0.510(4)	Fe1	0.490(4)
Ru2	0.457(4)	Fe2	0.543(4)

F. DFT Calculations. Optimised Cartesian coordinates for compounds (Å)

All complexes were optimized in the gas phase with scalar-relativistic DFT calculations using the ADF[i] 2019 program with all-electron ZORA-STO-TZ2P basis sets and the OLYP[ii,iii]-D3[iv] functional.

1. $RcCOCH_2COFc$

C	-2.082816000	1.671205000	-1.019978000
C	-1.790896000	1.428417000	0.358291000
C	-2.981942000	1.662864000	1.113141000
C	-4.012810000	2.052715000	0.199293000
C	-3.457069000	2.058352000	-1.119077000
H	-1.398369000	1.540392000	-1.846764000
H	-0.857588000	1.048487000	0.745383000
H	-3.095252000	1.518682000	2.178637000
H	-5.042346000	2.263043000	0.454854000
H	-3.992441000	2.275073000	-2.033385000
Fe	-3.286300000	0.231678000	-0.274922000
C	-2.624784000	-1.634054000	0.055367000
C	-3.848621000	-1.400319000	0.770871000
C	-4.856105000	-1.046889000	-0.172640000
C	-4.270494000	-1.063100000	-1.478054000
C	-2.894324000	-1.415794000	-1.344021000
H	-3.942057000	-1.442662000	1.845341000
H	-5.874506000	-0.766812000	0.059851000
H	-4.770271000	-0.805763000	-2.402160000
H	-2.179403000	-1.488013000	-2.150036000
C	-1.324377000	-1.856410000	0.712195000
O	-1.299654000	-2.113051000	1.951663000
C	-0.122353000	-1.717394000	-0.053027000
H	-0.174305000	-1.465706000	-1.101203000
C	1.109079000	-1.803222000	0.563572000
O	1.215635000	-2.090409000	1.859246000
H	0.243479000	-2.178310000	2.170484000
C	2.372003000	-1.566038000	-0.123718000
C	3.676043000	-1.652684000	0.483304000
C	4.645738000	-1.284531000	-0.497595000
C	3.955766000	-0.961337000	-1.710819000
C	2.555223000	-1.125819000	-1.484760000
H	3.861678000	-1.958949000	1.501987000
H	5.717212000	-1.255040000	-0.350152000
H	4.414933000	-0.648335000	-2.639182000
H	1.773430000	-0.969261000	-2.213481000
Ru	3.296533000	0.390090000	-0.131946000
C	2.150743000	2.233541000	-0.252671000
C	3.493001000	2.481034000	-0.689861000
C	4.374275000	2.199981000	0.405109000
C	3.576830000	1.780646000	1.518878000

C	2.205428000	1.801200000	1.111208000
H	1.252571000	2.363524000	-0.840237000
H	3.789507000	2.828042000	-1.670714000
H	5.452092000	2.293800000	0.394537000
H	3.944529000	1.496969000	2.495935000
H	1.360180000	1.524271000	1.725902000

2. $RcCOCH_2CORc$

C	-2.191814000	1.947796000	-1.054699000
C	-1.823265000	1.714977000	0.309625000
C	-2.991719000	1.882251000	1.122089000
C	-4.084497000	2.218039000	0.257574000
C	-3.590704000	2.258784000	-1.087818000
H	-1.529483000	1.901423000	-1.909061000
H	-0.840372000	1.435258000	0.659213000
H	-3.038976000	1.771878000	2.197130000
H	-5.102881000	2.412199000	0.566808000
H	-4.169675000	2.490939000	-1.971848000
Ru	-3.313363000	0.258334000	-0.278429000
C	-2.584253000	-1.740295000	0.057367000
C	-3.824390000	-1.575469000	0.766983000
C	-4.849210000	-1.294815000	-0.186675000
C	-4.256572000	-1.287368000	-1.491708000
C	-2.859994000	-1.556605000	-1.348510000
H	-3.928446000	-1.655035000	1.839044000
H	-5.893202000	-1.118142000	0.034918000
H	-4.775387000	-1.113224000	-2.425411000
H	-2.145320000	-1.640796000	-2.154378000
C	-1.276876000	-1.936893000	0.713575000
O	-1.245108000	-2.235956000	1.942081000
C	-0.082002000	-1.742678000	-0.050754000
H	-0.145771000	-1.444887000	-1.086296000
C	1.153957000	-1.844351000	0.550924000
O	1.276157000	-2.178710000	1.834327000
H	0.310389000	-2.283544000	2.151980000
C	2.407455000	-1.581711000	-0.143602000
C	3.719415000	-1.667451000	0.445841000
C	4.673663000	-1.272584000	-0.540064000
C	3.965941000	-0.934628000	-1.739318000
C	2.569443000	-1.115649000	-1.499191000
H	3.921827000	-1.988885000	1.456635000
H	5.746470000	-1.234891000	-0.404144000
H	4.410988000	-0.600149000	-2.667181000
H	1.777224000	-0.955106000	-2.215549000
Ru	3.311339000	0.381462000	-0.129454000
C	2.139172000	2.207656000	-0.206777000
C	3.473971000	2.484904000	-0.648875000
C	4.368182000	2.195438000	0.433846000
C	3.585933000	1.740973000	1.545029000
C	2.210919000	1.747465000	1.147946000

H	1.236137000	2.335448000	-0.786696000
H	3.756662000	2.856964000	-1.624858000
H	5.444120000	2.308134000	0.417326000
H	3.965076000	1.443310000	2.513365000
H	1.375659000	1.440295000	1.762315000

3. $RcCOCH_2COCH_3$

Ru	-1.438076000	0.061178000	-0.089947000
H	4.208883000	-0.910330000	-2.207946000
H	4.813023000	-1.973857000	-0.901944000
H	5.568444000	-0.399196000	-1.164319000
O	1.942908000	0.836703000	1.660293000
O	4.101941000	-0.319667000	1.040004000
C	0.286574000	1.353097000	0.058993000
C	-0.286108000	1.463810000	-1.261973000
C	-1.612513000	1.975679000	-1.125223000
C	-1.873890000	2.174007000	0.269203000
C	-0.710169000	1.791330000	1.000091000
C	-0.987447000	-2.038624000	-0.391062000
C	-2.145286000	-1.700489000	-1.162114000
C	-3.164798000	-1.260595000	-0.257942000
C	-2.640286000	-1.334748000	1.072809000
C	-1.293352000	-1.812150000	0.992428000
C	1.595761000	0.798525000	0.447591000
C	2.446402000	0.216374000	-0.558976000
C	3.664537000	-0.316200000	-0.215085000
C	4.611263000	-0.935117000	-1.192579000
H	0.206500000	1.229121000	-2.194210000
H	-2.300430000	2.177478000	-1.935956000
H	-2.797852000	2.544986000	0.692974000
H	-0.569143000	1.814900000	2.070616000
H	-0.042930000	-2.394460000	-0.780743000
H	-2.233706000	-1.760810000	-2.238816000
H	-4.158062000	-0.930754000	-0.532210000
H	-3.167048000	-1.068141000	1.979401000
H	-0.620481000	-1.965693000	1.825697000
H	2.129928000	0.184048000	-1.592509000
H	3.352971000	0.133475000	1.566091000

4. $[Ir(ppy)_2(CH_3COCHCOCH_3)]$ (1)

Ir	0.013316000	0.165256000	0.001025000
O	-0.628274000	1.734124000	1.365764000
O	0.652042000	1.727986000	-1.371193000
N	1.904026000	0.093662000	0.719982000
N	-1.879853000	0.108836000	-0.712585000
C	-0.566865000	2.981407000	1.118590000
C	-0.028071000	3.598696000	-0.026069000
H	-0.052647000	4.682615000	-0.039856000
C	0.540806000	2.977336000	-1.153852000

C	1.090278000	3.858619000	-2.260225000
H	0.678551000	3.530004000	-3.221377000
H	2.177847000	3.723292000	-2.312675000
H	0.867445000	4.917164000	-2.104119000
C	-1.143909000	3.865116000	2.208416000
C	2.336881000	0.794113000	1.786423000
H	1.589127000	1.413598000	2.266888000
C	3.644827000	0.718512000	2.238601000
H	3.950020000	1.302658000	3.101402000
C	4.540574000	-0.116870000	1.564351000
H	5.573328000	-0.199701000	1.893706000
C	4.094859000	-0.844997000	0.469732000
H	4.770014000	-1.504946000	-0.063476000
C	2.764095000	-0.740145000	0.045862000
C	2.138230000	-1.458662000	-1.050512000
C	2.814494000	-2.351919000	-1.896258000
H	3.879782000	-2.529904000	-1.772407000
C	2.122918000	-3.014467000	-2.903226000
H	2.644466000	-3.704557000	-3.561893000
C	0.748178000	-2.788887000	-3.060086000
H	0.203561000	-3.314073000	-3.843231000
C	0.071693000	-1.900911000	-2.225692000
H	-0.993684000	-1.745237000	-2.365367000
C	0.746548000	-1.203820000	-1.212345000
C	-2.312307000	0.814844000	-1.775617000
H	-1.560172000	1.422312000	-2.263958000
C	-3.624783000	0.756535000	-2.216721000
H	-3.928869000	1.343000000	-3.078418000
C	-4.527191000	-0.062703000	-1.531640000
H	-5.564556000	-0.129425000	-1.850477000
C	-4.082957000	-0.794546000	-0.439318000
H	-4.763642000	-1.441725000	0.102404000
C	-2.746727000	-0.709930000	-0.028777000
C	-2.123475000	-1.433449000	1.065360000
C	-2.806691000	-2.314963000	1.917506000
H	-3.875431000	-2.477021000	1.801668000
C	-2.117360000	-2.988073000	2.918506000
H	-2.644158000	-3.669929000	3.581508000
C	-0.737005000	-2.787781000	3.060474000
H	-0.193484000	-3.323481000	3.837205000
C	-0.054472000	-1.907197000	2.223078000
H	1.013970000	-1.767358000	2.354736000
C	-0.727504000	-1.197442000	1.217212000
H	-2.200881000	3.608117000	2.348471000
H	-0.633082000	3.649371000	3.154587000
H	-1.054867000	4.929829000	1.978849000

5. [Ir(ppy)$_2$(CH$_3$COCHCORc)], (2)

Ir	1.353179000	0.022494000	-0.268107000
Ru	-2.888453000	-3.457430000	-0.512978000

O	-0.707357000	-0.586430000	-0.169719000
O	0.973732000	0.657514000	-2.311327000
N	0.947190000	1.884514000	0.395769000
N	1.874311000	-1.843994000	-0.838683000
C	-1.494695000	-0.696077000	-1.167904000
C	-1.244357000	-0.260335000	-2.484570000
H	-2.044725000	-0.401467000	-3.199792000
C	-0.095945000	0.395146000	-2.957345000
C	-0.075487000	0.853117000	-4.402799000
H	0.746712000	0.349355000	-4.926729000
H	0.136025000	1.928256000	-4.433829000
H	-1.014881000	0.649129000	-4.922833000
C	-2.791423000	-1.330845000	-0.849333000
C	-3.770233000	-1.906506000	-1.738242000
H	-3.733376000	-1.896752000	-2.817873000
C	-4.800251000	-2.498127000	-0.941697000
H	-5.677669000	-3.008752000	-1.316409000
C	-4.467952000	-2.297498000	0.438234000
H	-5.052185000	-2.629783000	1.286232000
C	-3.230616000	-1.587211000	0.499302000
H	-2.691356000	-1.284315000	1.384614000
C	-2.075977000	-5.094747000	-1.691659000
H	-2.212737000	-5.205835000	-2.759256000
C	-2.929761000	-5.629144000	-0.672131000
H	-3.822805000	-6.218122000	-0.833678000
C	-2.387341000	-5.251856000	0.600064000
H	-2.800952000	-5.505172000	1.567423000
C	-1.199882000	-4.482427000	0.367249000
H	-0.564339000	-4.037518000	1.119457000
C	-1.009964000	-4.386645000	-1.049313000
H	-0.210370000	-3.858257000	-1.543663000
C	-0.271372000	2.291957000	0.798408000
H	-1.051098000	1.541721000	0.739681000
C	-0.502516000	3.576546000	1.262952000
H	-1.501451000	3.862286000	1.578123000
C	0.566928000	4.475904000	1.310421000
H	0.419114000	5.492059000	1.668143000
C	1.823786000	4.055437000	0.899760000
H	2.669287000	4.733162000	0.935447000
C	2.015283000	2.745496000	0.443324000
C	3.268990000	2.143000000	0.026578000
C	4.490262000	2.831952000	-0.033309000
H	4.540018000	3.889059000	0.215635000
C	5.647522000	2.164054000	-0.413934000
H	6.594699000	2.695606000	-0.463678000
C	5.583964000	0.799959000	-0.728649000
H	6.491890000	0.272871000	-1.017738000
C	4.373237000	0.111552000	-0.676786000
H	4.354461000	-0.945046000	-0.924234000
C	3.183555000	0.762041000	-0.314496000
C	2.110333000	-2.213377000	-2.112225000

H	1.993249000	-1.428880000	-2.849284000
C	2.486682000	-3.503151000	-2.449536000
H	2.665289000	-3.755363000	-3.490207000
C	2.631286000	-4.449395000	-1.430732000
H	2.924499000	-5.470236000	-1.661231000
C	2.396277000	-4.068695000	-0.118183000
H	2.509759000	-4.782098000	0.689822000
C	2.018598000	-2.754303000	0.179391000
C	1.796253000	-2.190380000	1.498471000
C	1.804691000	-2.936665000	2.686935000
H	1.955536000	-4.012895000	2.661051000
C	1.616980000	-2.300206000	3.907941000
H	1.619744000	-2.875154000	4.830491000
C	1.435267000	-0.910425000	3.941185000
H	1.300898000	-0.409174000	4.898692000
C	1.418227000	-0.164477000	2.764383000
H	1.267925000	0.909240000	2.816426000
C	1.575127000	-0.782620000	1.514815000

6. [Ir(ppy)$_2$(CH$_3$COCHCOFc)], (3)

Ir	1.191753000	0.001479000	-0.169095000
Fe	-3.322562000	-3.163222000	-0.344378000
O	-0.879636000	-0.567347000	0.019079000
O	0.759813000	0.628852000	-2.201844000
N	0.858410000	1.893887000	0.465142000
N	1.594729000	-1.898434000	-0.736137000
C	-1.719864000	-0.618164000	-0.941367000
C	-1.507924000	-0.177049000	-2.264880000
H	-2.348313000	-0.268908000	-2.941334000
C	-0.351360000	0.420759000	-2.794658000
C	-0.379033000	0.877979000	-4.240395000
H	0.414949000	0.362483000	-4.795102000
H	-0.152965000	1.950272000	-4.279893000
H	-1.341330000	0.689876000	-4.723382000
C	-3.033515000	-1.188686000	-0.577745000
C	-4.091261000	-1.662810000	-1.434088000
H	-4.100627000	-1.626811000	-2.513321000
C	-5.101920000	-2.250083000	-0.611333000
H	-6.004827000	-2.730763000	-0.963220000
C	-4.678012000	-2.151827000	0.752941000
H	-5.206194000	-2.544984000	1.611168000
C	-3.404741000	-1.507744000	0.777004000
H	-2.779477000	-1.323378000	1.637417000
C	-2.673373000	-4.548696000	-1.657171000
H	-2.800657000	-4.496200000	-2.729999000
C	-3.598607000	-5.123295000	-0.726372000
H	-4.546098000	-5.583751000	-0.971323000
C	-3.075247000	-4.926624000	0.592937000
H	-3.561604000	-5.208469000	1.516997000
C	-1.829019000	-4.228957000	0.476790000

H	-1.209692000	-3.879855000	1.290453000
C	-1.582472000	-3.999259000	-0.913063000
H	-0.753408000	-3.443628000	-1.319599000
C	-0.331513000	2.345621000	0.907159000
H	-1.126623000	1.609610000	0.928374000
C	-0.520430000	3.659917000	1.308093000
H	-1.497852000	3.979966000	1.655981000
C	0.562841000	4.543369000	1.247028000
H	0.447758000	5.581620000	1.548656000
C	1.791711000	4.076944000	0.798575000
H	2.647430000	4.740870000	0.748348000
C	1.940168000	2.738592000	0.410182000
C	3.161006000	2.092261000	-0.037565000
C	4.393995000	2.749816000	-0.180975000
H	4.478674000	3.814629000	0.021606000
C	5.519038000	2.039208000	-0.585581000
H	6.474225000	2.546046000	-0.699979000
C	5.412435000	0.664120000	-0.841580000
H	6.294915000	0.105295000	-1.149532000
C	4.189469000	0.006406000	-0.707825000
H	4.132855000	-1.058923000	-0.910694000
C	3.032117000	0.699404000	-0.316974000
C	1.738182000	-2.301096000	-2.013965000
H	1.673033000	-1.514225000	-2.755350000
C	1.948012000	-3.629511000	-2.351064000
H	2.050152000	-3.908686000	-3.395176000
C	2.015319000	-4.580538000	-1.327355000
H	2.165570000	-5.631969000	-1.558037000
C	1.888888000	-4.164445000	-0.009514000
H	1.946172000	-4.881541000	0.801284000
C	1.687025000	-2.810601000	0.287491000
C	1.594510000	-2.212502000	1.607189000
C	1.642100000	-2.939355000	2.807986000
H	1.716342000	-4.023873000	2.794238000
C	1.595792000	-2.271067000	4.027133000
H	1.630788000	-2.830290000	4.958775000
C	1.516628000	-0.870170000	4.046140000
H	1.495355000	-0.346346000	5.000668000
C	1.461307000	-0.142458000	2.857543000
H	1.393165000	0.940600000	2.895958000
C	1.476560000	-0.790283000	1.612039000

7. [Ir(ppy)$_2$(RcCOCHCORc)], (4)

Ir	1.953890000	0.015583000	-0.061391000
O	0.515697000	1.310781000	-1.013242000
O	0.690553000	-1.666755000	-0.561857000
N	1.024096000	0.305671000	1.713326000
N	2.984182000	-0.256715000	-1.780801000
C	0.129819000	-0.544258000	2.257603000
H	-0.077536000	-1.435329000	1.678278000

C	-0.490859000	-0.284167000	3.469979000
H	-1.215867000	-0.985911000	3.864393000
C	-0.181821000	0.900269000	4.146630000
H	-0.661394000	1.137132000	5.092796000
C	0.744531000	1.772141000	3.592304000
H	1.007376000	2.692447000	4.101426000
C	1.360106000	1.465181000	2.371293000
C	2.392639000	2.236203000	1.702864000
C	2.876238000	3.471845000	2.164328000
H	2.456136000	3.931332000	3.055690000
C	3.898284000	4.119129000	1.478412000
H	4.271466000	5.077725000	1.830090000
C	4.451298000	3.520136000	0.336496000
H	5.262033000	4.019163000	-0.192511000
C	3.973600000	2.295272000	-0.129223000
H	4.415661000	1.854736000	-1.017580000
C	2.921056000	1.631973000	0.523367000
C	2.735417000	0.406719000	-2.926940000
H	1.932719000	1.132561000	-2.875593000
C	3.461112000	0.176196000	-4.086177000
H	3.224246000	0.733223000	-4.987503000
C	4.485624000	-0.776242000	-4.059359000
H	5.074527000	-0.980103000	-4.950398000
C	4.747295000	-1.455711000	-2.876462000
H	5.542339000	-2.191336000	-2.828556000
C	3.989578000	-1.190816000	-1.727725000
C	4.165755000	-1.782913000	-0.412521000
C	5.093331000	-2.797024000	-0.124355000
H	5.722450000	-3.208508000	-0.909720000
C	5.208611000	-3.287753000	1.172117000
H	5.922886000	-4.076093000	1.396760000
C	4.402357000	-2.752098000	2.187211000
H	4.502065000	-3.125140000	3.205628000
C	3.473691000	-1.749346000	1.906612000
H	2.858717000	-1.354340000	2.709630000
C	3.314449000	-1.252710000	0.602386000
C	-0.619647000	0.929553000	-1.464279000
C	-1.109330000	-0.386352000	-1.520102000
H	-2.111604000	-0.507586000	-1.903834000
C	-0.473254000	-1.562057000	-1.082841000
C	-1.482606000	2.026398000	-1.958409000
C	-2.870039000	1.984212000	-2.356068000
H	-3.485970000	1.100490000	-2.434986000
C	-3.294094000	3.321454000	-2.639782000
H	-4.282774000	3.617606000	-2.965756000
C	-2.180712000	4.198535000	-2.419084000
H	-2.184487000	5.273008000	-2.547680000
C	-1.067520000	3.407059000	-1.996966000
H	-0.077630000	3.752297000	-1.737413000
C	-3.781743000	2.331565000	1.088598000
H	-4.382235000	1.433498000	1.035994000

C	-4.235767000	3.669503000	0.837219000
H	-5.240790000	3.959256000	0.559959000
C	-3.123996000	4.557070000	1.028485000
H	-3.142877000	5.633259000	0.916657000
C	-1.983506000	3.767071000	1.396892000
H	-0.987245000	4.135096000	1.601850000
C	-2.391792000	2.394425000	1.432959000
H	-1.753618000	1.555576000	1.659528000
C	-1.216138000	-2.839976000	-1.189293000
C	-0.844032000	-4.043492000	-0.491831000
H	0.031295000	-4.139057000	0.133611000
C	-1.810015000	-5.054160000	-0.789333000
H	-1.805577000	-6.069369000	-0.414703000
C	-2.786692000	-4.488376000	-1.674925000
H	-3.643852000	-5.002518000	-2.090091000
C	-2.429117000	-3.123492000	-1.918021000
H	-2.958205000	-2.438238000	-2.564056000
C	-3.734535000	-3.987431000	2.042711000
H	-3.707289000	-4.992192000	2.443120000
C	-4.731883000	-3.459207000	1.157168000
H	-5.589186000	-3.996131000	0.773105000
C	-4.395623000	-2.093332000	0.875218000
H	-4.954216000	-1.418077000	0.240717000
C	-3.192024000	-1.776934000	1.586007000
H	-2.677076000	-0.828860000	1.564427000
C	-2.781220000	-2.947930000	2.306631000
H	-1.908423000	-3.039184000	2.937630000
Ru	-2.792393000	-3.380396000	0.188504000
Ru	-2.660793000	3.167619000	-0.566542000

8. [Ir(ppy)$_2$(RcCOCHCOFc)], (5)

Ir	-1.535849000	-1.054752000	-0.126487000
O	0.439355000	-1.124062000	-0.993913000
O	-1.588235000	1.066602000	-0.552274000
N	-0.700678000	-0.758331000	1.693476000
N	-2.430756000	-1.444982000	-1.898664000
C	-0.568446000	0.443067000	2.289713000
H	-0.970181000	1.280566000	1.734657000
C	0.055577000	0.594760000	3.518093000
H	0.158808000	1.583745000	3.949473000
C	0.564528000	-0.539515000	4.158593000
H	1.071818000	-0.452122000	5.115851000
C	0.415308000	-1.780226000	3.554617000
H	0.792724000	-2.675057000	4.036410000
C	-0.235492000	-1.890948000	2.318581000
C	-0.533986000	-3.116335000	1.599414000
C	-0.151052000	-4.396771000	2.032681000
H	0.437702000	-4.520087000	2.938401000
C	-0.526925000	-5.519616000	1.303103000
H	-0.230391000	-6.511602000	1.634567000

C	-1.301499000	-5.362681000	0.143450000
H	-1.607978000	-6.242464000	-0.420480000
C	-1.681932000	-4.094076000	-0.294849000
H	-2.277717000	-3.999339000	-1.197355000
C	-1.293755000	-2.938806000	0.404345000
C	-1.768106000	-1.771395000	-3.025562000
H	-0.689862000	-1.817396000	-2.930826000
C	-2.424944000	-2.038103000	-4.217621000
H	-1.849613000	-2.297803000	-5.100947000
C	-3.821884000	-1.965960000	-4.245934000
H	-4.367668000	-2.172466000	-5.163381000
C	-4.505851000	-1.631702000	-3.084108000
H	-5.588786000	-1.577581000	-3.078695000
C	-3.801835000	-1.371292000	-1.900894000
C	-4.369538000	-1.051590000	-0.602294000
C	-5.740236000	-0.852676000	-0.370461000
H	-6.453070000	-0.917300000	-1.188891000
C	-6.194679000	-0.559855000	0.911066000
H	-7.254584000	-0.398476000	1.091479000
C	-5.276079000	-0.481779000	1.968270000
H	-5.632162000	-0.265222000	2.974617000
C	-3.912390000	-0.674813000	1.744516000
H	-3.221553000	-0.606198000	2.579493000
C	-3.419542000	-0.943491000	0.456964000
C	1.095351000	-0.100979000	-1.393778000
C	0.647114000	1.230452000	-1.430043000
H	1.362390000	1.966169000	-1.766532000
C	-0.599807000	1.733490000	-1.016869000
C	2.476516000	-0.391982000	-1.840145000
C	3.532878000	0.529629000	-2.186605000
H	3.448258000	1.604303000	-2.253159000
C	4.719590000	-0.227771000	-2.445153000
H	5.680599000	0.178018000	-2.733582000
C	4.411539000	-1.616221000	-2.258049000
H	5.102049000	-2.440945000	-2.377217000
C	3.035861000	-1.720309000	-1.882882000
H	2.483305000	-2.620516000	-1.657563000
C	4.374391000	0.779470000	1.289059000
H	4.291221000	1.857253000	1.250698000
C	5.566329000	0.012465000	1.064786000
H	6.543812000	0.410179000	0.825381000
C	5.236324000	-1.375347000	1.224131000
H	5.920605000	-2.207465000	1.122722000
C	3.840447000	-1.465171000	1.546218000
H	3.279077000	-2.373032000	1.720813000
C	3.311276000	-0.134624000	1.585453000
H	2.284753000	0.125542000	1.787502000
C	-0.813108000	3.197538000	-1.071185000
C	-1.841479000	3.883762000	-0.338567000
H	-2.573028000	3.394624000	0.286527000
C	-1.688082000	5.286237000	-0.551840000

H	-2.284195000	6.067410000	-0.099906000
C	-0.567186000	5.482482000	-1.420803000
H	-0.171240000	6.435633000	-1.744730000
C	-0.020131000	4.200459000	-1.735737000
H	0.846512000	4.020310000	-2.354052000
C	0.267846000	5.454912000	1.993851000
H	-0.384075000	6.220547000	2.391965000
C	1.378826000	5.669564000	1.116703000
H	1.712671000	6.625708000	0.737177000
C	1.929529000	4.390861000	0.779709000
H	2.751617000	4.210302000	0.100931000
C	1.161080000	3.387338000	1.448762000
H	1.289979000	2.323256000	1.335769000
C	0.132897000	4.043250000	2.198972000
H	-0.642302000	3.562430000	2.777562000
Fe	-0.008279000	4.497091000	0.244339000
Ru	4.066192000	-0.547954000	-0.397508000

9. [Ir(ppy)$_2$(FcCOCHCOFc)], (6)

Ir	1.720501000	0.258840000	-0.139217000
O	0.035630000	1.293458000	-1.001305000
O	0.714577000	-1.614438000	-0.541999000
N	0.839042000	0.436139000	1.672884000
N	2.682864000	0.134717000	-1.914234000
C	0.135926000	-0.537780000	2.283959000
H	0.101744000	-1.482782000	1.757778000
C	-0.519903000	-0.331006000	3.487418000
H	-1.095989000	-1.134902000	3.930294000
C	-0.448312000	0.931329000	4.084516000
H	-0.968539000	1.131374000	5.017549000
C	0.296316000	1.928145000	3.470169000
H	0.375939000	2.911299000	3.919684000
C	0.957183000	1.672578000	2.262141000
C	1.826222000	2.581261000	1.536815000
C	2.106060000	3.896468000	1.942746000
H	1.630891000	4.314529000	2.826727000
C	3.000261000	4.673400000	1.214135000
H	3.218283000	5.692263000	1.524401000
C	3.628336000	4.128373000	0.084155000
H	4.339746000	4.731610000	-0.477929000
C	3.349633000	2.825613000	-0.328737000
H	3.843437000	2.428325000	-1.210352000
C	2.429652000	2.026552000	0.368619000
C	2.253143000	0.718944000	-3.049703000
H	1.338431000	1.292246000	-2.957583000
C	2.938994000	0.595495000	-4.248727000
H	2.553974000	1.081581000	-5.139902000
C	4.116998000	-0.158841000	-4.274388000
H	4.678219000	-0.276176000	-5.198112000

C	4.565565000	-0.753681000	-3.102214000
H	5.479939000	-1.336350000	-3.095425000
C	3.841398000	-0.602507000	-1.912362000
C	4.192465000	-1.131199000	-0.605639000
C	5.304367000	-1.953908000	-0.362510000
H	5.961778000	-2.247623000	-1.176990000
C	5.566890000	-2.407248000	0.925954000
H	6.423823000	-3.048700000	1.115970000
C	4.720132000	-2.026801000	1.977442000
H	4.929960000	-2.373597000	2.988336000
C	3.613511000	-1.210352000	1.742864000
H	2.974190000	-0.928941000	2.574400000
C	3.309898000	-0.753814000	0.449970000
C	-1.049229000	0.728105000	-1.378573000
C	-1.320343000	-0.650448000	-1.395888000
H	-2.310876000	-0.938652000	-1.715572000
C	-0.481712000	-1.703487000	-0.986818000
C	-2.110868000	1.660432000	-1.811543000
C	-3.499125000	1.385771000	-2.085091000
H	-3.972506000	0.415434000	-2.076318000
C	-4.162868000	2.629102000	-2.319422000
H	-5.219649000	2.755747000	-2.513422000
C	-3.199141000	3.679509000	-2.186278000
H	-3.402240000	4.739280000	-2.260212000
C	-1.939433000	3.088588000	-1.868633000
H	-1.012629000	3.597506000	-1.651162000
C	-4.791690000	2.806293000	0.852670000
H	-5.841237000	2.908643000	0.612137000
C	-3.844485000	3.875932000	0.957536000
H	-4.053783000	4.926170000	0.805536000
C	-2.559631000	3.304037000	1.232257000
H	-1.623787000	3.838121000	1.316086000
C	-2.715127000	1.884980000	1.297590000
H	-1.914715000	1.176251000	1.425244000
C	-4.091403000	1.574178000	1.062863000
H	-4.516828000	0.582058000	1.012403000
C	-1.028023000	-3.078692000	-1.019011000
C	-0.487634000	-4.171190000	-0.258336000
H	0.383118000	-4.099885000	0.375699000
C	-1.317755000	-5.314149000	-0.458327000
H	-1.195974000	-6.279425000	0.013993000
C	-2.375823000	-4.942923000	-1.348477000
H	-3.190186000	-5.578334000	-1.669594000
C	-2.207349000	-3.565313000	-1.688187000
H	-2.859726000	-2.988817000	-2.326986000
C	-3.134556000	-4.442550000	2.043842000
H	-2.962315000	-5.426579000	2.457819000
C	-4.189896000	-4.080697000	1.146575000
H	-4.954385000	-4.743725000	0.765200000
C	-4.016824000	-2.703173000	0.793770000
H	-4.627279000	-2.143425000	0.098781000

C	-2.856691000	-2.214328000	1.472563000
H	-2.430653000	-1.231463000	1.356307000
C	-2.309909000	-3.288567000	2.244669000
H	-1.405670000	-3.255822000	2.834886000
Fe	-2.389413000	-3.777812000	0.294250000
Fe	-3.307828000	2.586762000	-0.492279000

([i]) Velde, G. T.; Bickelhaupt, F. M.; Baerends, E. J.; Guerra, C. F.; van Gisbergen, S. J. A.; Snijders, J. G.; Ziegler, T. Chemistry with ADF. *J. Comput. Chem*. **2001**, *22*, 931-967.

([ii]) Handy, N. C.; Cohen, A. J. Left-right correlation energy. *Mol. Phys*. 2001, *99*, 403-412.

([iii]) Lee, C.; Yang, W.; Parr, R. G. Development of the Colle-Salvetti correlation-energy formula into a functional of the electron density. *Phys. Rev. B* **1988**, *37*, 785-789.

([iv]) Grimme, S.; Anthony, J.; Ehrlich, S.; Krieg, H. A Consistent and Accurate *Ab Initio* Parametrization of Density Functional Dispersion Correction (DFT-D) for the 94 Elements H-Pu. *J. Chem. Phys*. **2010**, *132*, Art. no. 154104.
